# Resistance of the cold-water coral *Dendrophyllia cornigera* to single and combined global change stressors

**DOI:** 10.1038/s41598-025-24028-1

**Published:** 2025-11-17

**Authors:** Cristina Gutiérrez-Zárate, Andrea Gori, Alfredo Veiga, Marta Álvarez, César González-Pola, Maria Rakka, Juancho Movilla, Kristina K. Beck, Eva M. S. Atienza, Rubén Acerbi, Covadonga Orejas

**Affiliations:** 1 Centro Oceanográfico de Gijón, Spanish Institute of Oceanography, IEO-CSIC, Av. Príncipe de Asturias, 70 Bis, Gijón, 33212 Spain; 2 Departament de Biologia Evolutiva, Universitat de Barcelona, Ecologia i Ciències Ambientals, Av. Diagonal 643, Barcelona, 08028 Spain; 3 Aquarium Finisterrae, Concello da Coruña, Paseo Marítimo Alcalde Francisco Vázquez, 34, A Coruña, 15001 Spain; 4 Institut de Recerca de la Biodiversitat (IRBio), Universitat de Barcelona, Barcelona, 08028 Spain; 5 Centro Oceanográfico de A Coruña, Spanish Institute of Oceanography, IEO-CSIC, Paseo Marítimo Alcalde Francisco Vázquez, 10, A Coruña, 15010 Spain; 6 Dalhousie University, Halifax, NS B3H 4R2 Canada; 7 Centro Oceanográfico de Baleares, Spanish Institute of Oceanography, IEO-CSIC, Estació d’Investigació Jaume Ferrer, Maó, 07700 Spain; 8 School of Geosciences, University of Edinburgh, Edinburgh, UK

**Keywords:** Climate-change ecology, Ecophysiology, Marine biology

## Abstract

Current knowledge of the consequences on global change in deep marine ecosystems is still limited, especially since environmental pressures do not act separately, and their potential interactions are mostly unknown. Cold-water corals (CWC) play a significant role in the deep sea, being ecosystem engineers supporting high biodiversity. However, global change may impact CWCs, compromising their integrity and survival. In this study, a nine-month aquaria experiment was conducted on the CWC *Dendrophyllia cornigera* from the NW Iberian Shelf (NE Atlantic Ocean). The aim was to assess the individual and combined effects of elevated temperature (12 vs. 15 °C), low pH (~ 7.99 vs. 7.69 pH_T_) and low oxygen (~ 6.4 vs. 4.7 mL L^−1^), based on the IPCC RCP 8.5 scenario. During the experiment, coral survival, skeletal growth, tissue cover and respiration were monitored as response variables. No significant effects were found on any of the response variables for either individual or combined stressors, pointing to the resistance of *D. cornigera* to different global change scenarios. Such a physiological resistance may support *D. cornigera* persistence under future conditions where other CWCs with narrower tolerance ranges may face greater limitations. However, further research is needed to assess potential trade-offs to cope with environmental change, which might impact the long-term survival capacity of this species.

**Supplementary Information:**

The online version contains supplementary material available at 10.1038/s41598-025-24028-1.

## Introduction

Cold-water corals (CWC) are among the main ecosystem engineers (*sensu* Jones et al.^1^) in the deep sea, forming complex frameworks that support a high diversity of associated fauna^2–5^. However, CWCs are facing growing threats due to the increasing pressure of global change, including ocean warming, acidification and deoxygenation^6,7^.

Under the increasing atmospheric CO_2_ concentration, ocean warming is not restricted to shallow depths, but water temperatures at greater depths are also increasing, and are expected to further increase at higher rates^8^ with temperature rising up to 4 °C in depths ranging from 200 to 3,000 m by the end of the century^9^. Temperature is a major factor controlling the distribution of CWCs^7,10,11^ and ocean warming may consequently push them beyond their physiological limits (e.g.^12–14^), and shift species’ distributions^7^. This can be of special concern, as warming can occur in combination with Ocean Acidification (OA), which increases total dissolved inorganic carbon (DIC) and partial pressure of CO_2_ (*p*CO_2_), and decreases pH and calcium carbonate saturation states (Ω)^15^. Ocean acidification extends to the deep ocean^16^, with a projected decrease of up to 0.37 pH units by the end of the century^9^. Such decrease poses a threat to calcifying organisms like CWCs^17–20^ and to the ecosystems that they sustain^21,22^. In addition, ocean warming and OA in the deep sea are occurring under a projected decline in dissolved oxygen (DO) concentrations because of their lower solubility in warmer water, as well as an increased stratification and reduced ventilation in the intermediate and deep ocean layers^23^. End-of-the-century projections estimate a decrease of DO at the deep seafloor of 0.05–0.24 mL L^−1^^9,24^. While CWCs have been found in natural hypoxic waters (e.g^25–29^), their response to deoxygenation remains mostly unknown (but see^14,30,31^).

Global-change stressors happening simultaneously may have cumulative effects^32–34^. Given the complex responses of organisms to combinations of stressors, there is a growing need to develop multiple-stressor experiments to assess the potential impacts of global change^35^. Further, these should be regionally targeted to be based on realistic projections and encompass the regional ecological conditions^35^. Very few multiple-stressor experiments have been conducted with CWCs^12,17–19,36,37^, given their difficult access and the technical complexity in their maintenance and experimentation^38^. Moreover, due to their slow growth and reduced metabolism, some responses of CWCs to global change might be only detectable after long-term exposures^12,20,37,39^. Nevertheless, despite some experiments showing the CWC resilience to OA (e.g^40–42^), there is growing evidence of CWC vulnerability to potential interactions of stressors associated to the ongoing global change (e.g^12,36,37^). Still, no experimental study has addressed the potential interactions among warming, OA and deoxygenation on CWCs.

Here, we investigate the single and cumulative impacts of elevated temperature, low pH and low oxygen on the scleractinian CWC *Dendrophyllia cornigera* in a long-term aquaria experiment. Although previous studies have revealed its resistance to elevated temperature^39,43,44^ and low pH^20,45^, its response to low oxygen and the potential interactions among stressors is unknown. By analysing coral survival, skeletal growth, tissue cover and respiration, we aim to increase the current knowledge on how CWC will cope with environmental conditions projected for the end of the century.

## Results

### Experimental conditions

Target temperature, pH and DO were mostly met for each treatment throughout the experimental time (Table 1), with no significant effects on the dissolved inorganic nutrient concentrations and prokaryotic abundances among the treatments. Low-temperature treatments displayed slightly higher values (~ 0.13 °C) than the 12 °C target. Low-pH treatments were, on average, 0.02 pH_T_ units below the 7.69 target. Low DO treatments were maintained ~ 0.03 mg L^−1^ above the 4.7 mg L^−1^ target. For more details of the experimental conditions, see Gutiérrez-Zárate et al.^46^.

**Table 1:** In situ values for the measured water parameters of the experimental aquaria for each treatment during the 9 months of the experiment. Temperature and dissolved oxygen concentration (DO) were measured five times per week, pH (total scale) was measured every 1–2 months and salinity was measured weekly. Mean values for the concentration of silicate and phosphate in the aquaria experiments were used in the calculations (2.1 and 0.31 µmol Kg^−1^, respectively). TA = total alkalinity; *p*CO_2_ = partial pressure of CO_2_; DIC = dissolved inorganic carbon; HCO_3_^−^ = bicarbonate ion concentration; CO_3_^2−^ = carbonate ion concentration; Ω_Ca_ = saturation state of seawater with respect to calcite; Ω_Ar_ = saturation state of seawater with respect to aragonite.

Treatment	Temperature (ºC)	pH	DO (mL L^−1^)	Salinity	TA (µmol kg^−1^)	pCO_2_ (µatm)	DIC (µmol kg^−1^)	HCO_3_^−^ (µmol kg^−1^)	CO_3_^2−^ (µmol kg^−1^)	Ω_Ca_	Ω_Ar_
Control	12.36 ± 0.43	7.98 ± 0.04	6.42 ± 0.13	35.0 ± 0.5	2,332 ± 32	483 ± 51	2,160 ± 31	2,011 ± 32	129 ± 10	3.08 ± 0.23	1.97 ± 0.15
↑Temp	15.07 ± 0.21	7.94 ± 0.04	6.29 ± 0.13	35.1 ± 0.5	2,333 ± 30	546 ± 54	2,162 ± 31	2,011 ± 33	130 ± 10	3.10 ± 0.22	1.99 ± 0.15
↓pH	12.42 ± 0.44	7.69 ± 0.08	6.40 ± 0.14	35.1 ± 0.5	2,333 ± 30	1,013 ± 171	2,266 ± 38	2,153 ± 40	72 ± 13	1.72 ± 0.30	1.10 ± 0.19
↓DO	12.28 ± 0.33	8.00 ± 0.04	4.75 ± 0.23	35.0 ± 0.4	2,333 ± 30	462 ± 54	2,153 ± 32	2,000 ± 35	134 ± 10	3.18 ± 0.24	2.04 ± 0.15
↑Temp x ↓pH	15.00 ± 0.24	7.68 ± 0.04	6.24 ± 0.11	35.1 ± 0.4	2,333 ± 30	1,061 ± 100	2,261 ± 35	2,145 ± 35	76 ± 6	1.80 ± 0.14	1.16 ± 0.09
↑Temp x ↓DO	15.01 ± 0.30	7.95 ± 0.04	4.77 ± 0.19	35.1 ± 0.5	2,333 ± 30	527 ± 60	2,156 ± 34	2,003 ± 38	133 ± 10	3.17 ± 0.24	2.04 ± 0.16
↓pH x ↓DO	12.16 ± 0.33	7.67 ± 0.05	4.76 ± 0.25	35.1 ± 0.4	2,333 ± 30	1,072 ± 133	2,276 ± 34	2,165 ± 34	67 ± 7	1.60 ± 0.17	1.03 ± 0.11
↑Temp x ↓pH x ↓DO	15.04 ± 0.30	7.64 ± 0.03	4.81 ± 0.30	35.1 ± 0.5	2,333 ± 30	1,159 ± 89	2,272 ± 35	2,159 ± 34	70 ± 4	1.67 ± 0.08	1.07 ± 0.05

### Coral survival

Over the 9 months of experimental time, coral survival was high, with an overall average survival of 95.8% across a total of 110 polyps. Survival only slightly decreased in the low oxygen (88.9%), combined low pH and low oxygen (89.0%), and combined elevated temperature, low pH, and low oxygen (88.9%) treatments, where three polyps with severe tissue necrosis died (Supplementary Fig. S1). Therefore, these nubbins were excluded from further statistical analyses.

### Coral skeletal growth and tissue cover

**Fig. 1:**
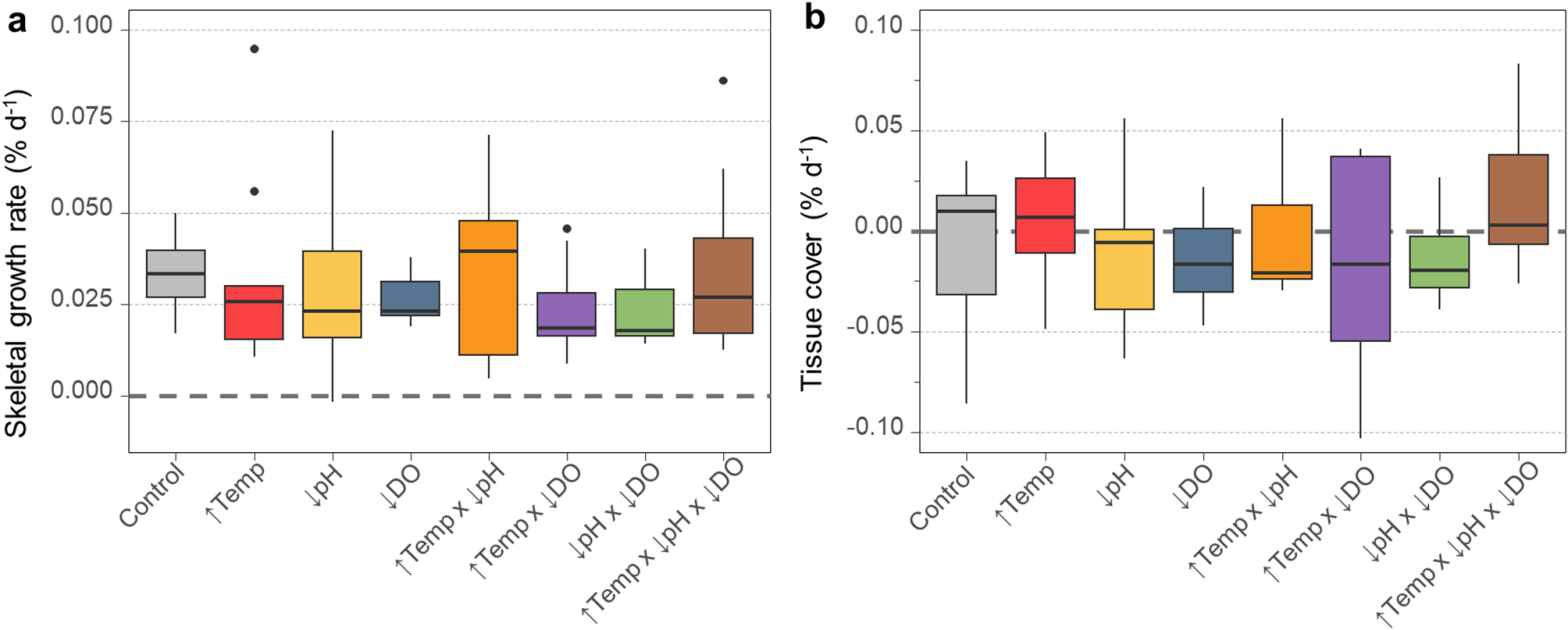
Coral skeletal growth (**a**) and tissue cover (**b**) rates (both in % d^−1^) of *Dendrophyllia cornigera* in response to single and combined global change stressors over 9 months. Horizontal dashed lines indicate the value of 0% d^−1^. Treatment conditions are: control (grey), elevated temperature (↑Temp, red), low pH (↓pH, yellow), low oxygen (↓DO, blue), combined elevated temperature and low pH (↑Temp x ↓pH, orange), combined elevated temperature and low oxygen (↑Temp x ↓DO, purple), combined low pH and low oxygen (↓pH x ↓DO, green), and combined elevated temperature, low pH, and low oxygen (↑Temp x ↓pH x ↓DO, brown). Note the different scales of the Y axes.

Skeletal growth rates ranged from < 0.01 to 0.09% d^−1^ across all treatments with an average of 0.03 ± 0.02% d^−1^ (mean ± SD) (Fig. 1a, Supplementary Table S1). None of the individual or combined effects of elevated temperature, low pH and low oxygen significantly affected the skeletal growth rates of *D. cornigera* (Linear Mixed-Effects Model, LMM, *p* = 0.77, Supplementary Table S2, Supplementary Fig. S2), as shown by the overlapping of the 95% CI among treatments (Supplementary Fig. S3).

The overall change in tissue cover ranged from − 0.1 to 0.08% d^−1^, with an average of −0.01 ± 0.04% d^−1^ (mean ± SD) (Fig. 1b, Supplementary Table S1). As the tissue from the nubbins covered most of the skeletal surface at the beginning of the experiment, tissue cover values around 0 indicate that the nubbins maintained the tissue area covering their skeleton over the 9 months of the experiment. Tissue cover of the nubbins did not differ significantly between treatments (LMM, *p* = 0.58, Supplementary Table S3), as further demonstrated by the overlap of the 95% CI among treatments (Supplementary Fig. S4).

### Coral respiration

**Fig. 2:**
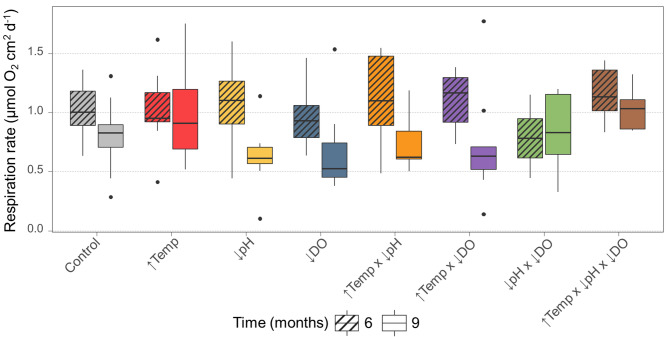
Respiration rates of *Dendrophyllia cornigera* in response to single and multiple global change stressors after 6 and 9 months (stripped and blank patterns, respectively). Respiration rates were normalised to the tissue surface area at each measurement time. Treatment conditions are: control (grey), elevated temperature (↑Temp, red), low pH (↓pH, yellow), low oxygen (↓DO, blue), combined elevated temperature and low pH (↑Temp x ↓pH, orange), combined elevated temperature and low oxygen (↑Temp x ↓DO, purple), combined low pH and low oxygen (↓pH x ↓DO, green), and combined elevated temperature, low pH, and low oxygen (↑Temp x ↓pH x ↓DO, brown).

After 6 months, respiration rates ranged from 0.41 to 1.62 µmol O_2_ cm^2^ d^−1^ across all treatments, with an average value of 1.03 ± 0.29 µmol O_2_ cm^2^ d^−1^. After 9 months, the average respiration was 0.81 ± 0.35 µmol O_2_ cm^2^ d^−1^, ranging from 0.10 to 1.78 µmol O_2_ cm^2^ d^−1^ (Fig. 2, Supplementary Table S1). Although respiration rates did not differ significantly between treatments (LMM, *p* = 0.062), we found an interaction between treatment and time (LMM, *p* = 0.029, Supplementary Table S4). Respiration rates tended to decrease in most treatments and generally declined over time (despite overlapping 95% CI among treatments after 6 and 9 months, Supplementary Fig. S5), with statistically significant decreases in the low pH (*p* < 0.001), low oxygen (LMM, *p* = 0.018), combined elevated temperature and low pH (LMM, *p* = 0.002), and combined elevated temperature and low oxygen (LMM, *p* = 0.002) treatments (Supplementary Table S5).

## Discussion

For the first time, we assessed the individual and combined effects of elevated temperature, low pH and low oxygen on a CWC species. Overall, no significant impacts were detected on the measured response variables (survival, skeletal growth, tissue cover and respiration) in any of the treatments over the whole duration of the experiment. However, a decrease in respiration rates from month 6 to the end of the experiment was statistically significant in several treatments (low pH, low oxygen, combined elevated temperature and low pH, and combined elevated temperature and low oxygen).

When individually exposed to elevated temperature or low pH, none of the measured response variables were affected. These results are in line with previous studies assessing the effects of elevated temperature^39,43,44^ and low pH^20,45^ on Mediterranean specimens of the species. Therefore, our results suggest that Atlantic specimens of *D. cornigera* share a similar tolerance to increased temperature and reduced pH compared to their Mediterranean counterparts.

The resistance of *D. cornigera* to warm temperatures is known, as the species occurs off Madeira (16.6 °C^47,48^) and Canary Islands (17 °C^49,50^) in the Atlantic Ocean. This is consistent with the thermal tolerance displayed by Mediterranean *D. cornigera* in aquaria at 16 ºC^43^ and 17.5 ºC^44^. These findings confirm the ability of this species to cope with relatively high temperatures, compared to other CWCs. For example, this contrasts with the 15 °C thermal limit observed for the CWC *Desmophyllum pertusum* (synonym *Lophelia pertusa*^51^) from the Mediterranean Sea^52^, the NE Atlantic^18,53^ and the Gulf of Mexico^13,14^ as well as for *Caryophyllia huinayensis* from Chilean fjords^12^. Moreover, fossil records showed that dendrophylliid corals dominated CWC communities in the Mediterranean Sea^54,55^ in the warmer period from the Miocene to the Early Pleistocene (8–7 Ma, sea surface temperatures of ~ 26 °C during the Late Pliocene^56,57^). Conversely, after the Pliocene-Pleistocene transition (~ 2.8 Ma), climatic oscillations and cooling events resulted in a decrease in dendrophylliid abundance and diversity, with a shift to *D. pertusum*-dominated CWC communities^54,55^. Similarly, the dominance of dendrophylliid in CWC paleocommunities in the Gulf of Cádiz (45.8–48 kyr BP) was associated with warm periods and relatively stable conditions^58^. All these experimental findings, as well as present and past observations of species distribution, support the hypothesis of the persistence, and even expansion, of *D. cornigera* in regions or depths where other CWC species, which are more constrained by their upper-thermal limits, might disappear under future ocean warming^47^.

The projected pH conditions for the study area, as tested in our experiment, resulted in aragonite oversaturation values (Ω_Ar_ > 1) (Table 1). This had no detectable impact on the corals, nor on the exposed skeleton as previously observed for *D. pertusum*^37^. The threshold between the calcification and skeletal dissolution (positive and negative skeletal growth rates, respectively) has been found at a lower Ω_Ar_ than those found in our experimental low-pH treatments for other CWC species. For example, skeletal dissolution was detected in *Madrepora oculata* at Ω_Ar_ = 0.92^59^, whereas it was at Ω_Ar_ = 0.80 for *Desmophyllum dianthus*^19^. Therefore, coral skeleton dissolution for *D. cornigera* at the sampling site might occur at *p*CO_2_ levels higher than the simulated 1,000 ppm (7.69 pH_T_ units).

Similarly to OA, projected deoxygenated conditions for the study area (4.7 mL L^−1^) did not significantly affect any of the measured response variables. This level of DO is consequently above the limiting concentration for our target species, at least in the time frame of our experiment. This is also corroborated by the occurrence of the species in the Sicilian Channel where DO is even lower (3.75–3.84 mL L^−1^ DO^60^). The hypoxia threshold varies among marine benthic taxa^61^ and is highly species-specific among tropical corals^62^. In CWCs, the hypoxia threshold has only been assessed for NE Atlantic *D. pertusum*, which displayed a decrease in respiration when DO falls below 3 mL L^−1^^30^. However, *D. pertusum* has also been observed thriving in hypoxic waters along the Angola margin (< 1 mL L^−1^ DO^27^) and the Gulf of Mexico (2.53 mL L^−1^^25^), suggesting the existence of additional mechanisms that allow its persistence even under low DO^31^.

The combination of several pressures may uncover hidden impacts that cannot be detected when only investigating their individual effects. For example, Gori et al.^36^ found no impacts of the single effects of elevated temperature or low pH on the respiration rates of the CWC *D. dianthus*, however, when combined, respiration significantly decreased. Our results showed no significant synergistic nor antagonistic effect among any of the explored global change stressors on the measured response variables, further supporting the resistance of *D. cornigera* to the forecasted conditions driven by global change in the study area. The wide geographical and bathymetrical distribution of the species^43,47^, and its significant trophic plasticity^63–65^, allow *D. cornigera* to grow under a wide range of environmental settings (30–1,200 m depth, ~ 7–17 °C^43,47^), with a potential acclimatisation capability of the species to highly changing conditions or adaptive local genetic changes. However, knowledge on CWC population genetics remains very limited^66^, including for *D. cornigera* populations and their genetic structure, which might determine potential local adaptations to environmental conditions across different sites.

Coral respiration displayed a non-significant, but overall negative trend for most experimental treatments. A similar decrease in respiration has been already observed in *D. pertusum* maintained under elevated temperatures after 12 months^37^. This decline, coupled with no significant effects on calcification rates, was interpreted by Hennige et al.^37^ as the result of potential changes in the energetic pathways. Previously, Büscher et al.^18^ found a general decrease in the respiration rates of *D. pertusum* over a 13-month experiment. The authors attributed this result to an “aquaria effect” most likely due to lower food quality compared to in situ conditions. Alongside the decreasing trend of respiration in our study, the skeletal growth rates were lower than those previously reported (0.04–0.06% d^−1^^43,67^). Collectively, these results point towards a low supply or quality of food for the corals in our experiment compared to the natural conditions.

It has been previously shown that CWCs decrease their metabolism under zooplankton exclusion^68^. Limited food availability may also explain why we found no increasing skeletal growth rates under warmer conditions (15 °C), contrary to previously reported for Mediterranean specimens under aquaria conditions at 16–17.5 °C^39,43,44^ (Supplementary Table S6). Although *Artemia salina* nauplii were previously used in experiments with this species^45,63,67,69,70^, several studies included larger food items (*Mysis*) in their diet^20,38,39,43,63,67^. *Mysis* supply a higher carbon content than *A. salina* nauplii (783.2 ± 36.6 and 47.2 ± 12.6 µg C polyp^−1^ h^−1^, respectively)^63^. Therefore, the selected food diet for the experiment may not have been optimal for this species. The often-documented resistance to environmental stress of corals in aquaria experiments has been frequently attributed to overfeeding^68,71,72^, with some CWCs showing increased food intake to maintain metabolic functions under stress^52,73^. Nevertheless, our study shows that, even under potentially low food supply, the measured response variables on *D. cornigera* appear to be unaffected by global change stressors, neither individually nor in combination, over the investigated time period of 9 months.

Although *D. cornigera* displayed remarkable capacity to withstand environmental changes in the present study, CWCs from different geographic locations may exhibit contrasting responses, with adaptation or acclimatisation to local environmental settings possibly playing a crucial role in species vulnerability to global change. For example, Georgian et al.^73^ found potential regional differences in the ecophysiological response of *D. pertusum* to OA. In their study, corals from the Gulf of Mexico significantly decreased their respiration and prey capture rates under OA conditions, while those from Tisler Reef (Norway) displayed increased rates of both response variables (Supplementary Table S6). Therefore, the wide geographic distribution of *D. cornigera* warrants future investigation into potential differential responses of this species to global change across its occurrence sites. The adaptation and/or acclimatisation to the local oceanographic variability has already been observed in CWC habitats exposed to short-term and seasonal changes derived from tidal effects^74,75^, meteorological events^74,76^, or water mass upwelling and intrusions^77,78^. Adaptation or acclimatisation to such events might potentially contribute to enhanced coral resistance to global change^13,79–82^. Nevertheless, potential trade-offs may involve the observed resistance of *D. cornigera* to global change, possibly affecting species reproduction^83^ or vulnerability during early-life stages^12,19,84^. This knowledge is key to determine if prolonged exposures to forecasted conditions driven by global change may lead to changes in population dynamics and viability of this CWC species.

## Conclusions

This aquaria study demonstrates the capability of *D. cornigera* to cope with the single and simultaneous effects of elevated temperature, low pH and low oxygen, based on global change projections for the study area (NW Iberian Peninsula) over a time period of 9 months. No single or interactive effect of any stressor was found in any of the treatments and response variables. However, these findings raise new questions about the potential mechanisms and possible trade-offs that provide the species with the observed resistance. While the duration of this study exceeds that of most ecophysiological experiments on CWCs conducted in aquaria, it cannot reflect ecologically relevant timescales, as the impacts of global change may persist and intensify over years or even decades (see IPCC^85^).

## Methods

### Sampling site

**Fig. 3:**
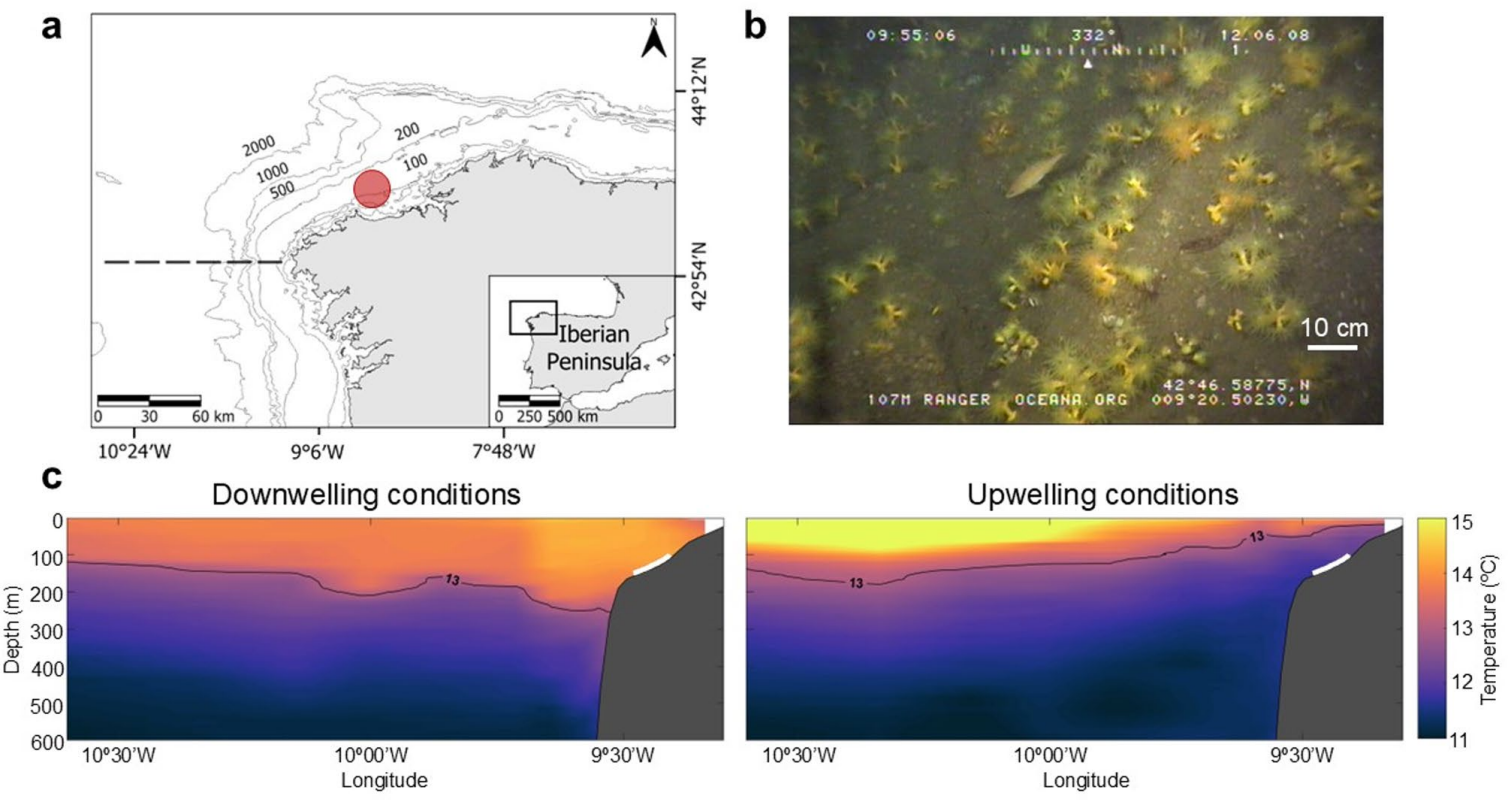
Sampling site. (**a**) Location of the collection site (red spot), dashed line indicates the location of the vertical sections (**c**); (**b**) Aggregations of *Dendrophyllia cornigera* colonies close to the sampling site (Image credit: ©OCEANA); (**c**) Vertical section of seawater temperature (ºC) close to the sampling site during downwelling (left) and upwelling (right) conditions, white lines indicate the depth at which *D. cornigera* occurs at the sampling site. Temperature data based on RADPROF cruises^89^.

*Dendrophyllia cornigera* known distribution ranges from 30 to 1,200 m depth and encompasses the NE Atlantic, from the Celtic Sea to the Cabo Verde archipelago, and the Mediterranean Sea^43,47,86^. In the NW Iberian shelf (NE Atlantic), it forms dense aggregations at circalittoral and bathyal depths (Fig. 3a, b). This area is located in the northern edge of the North East Atlantic Upwelling System^87^ and is subjected to strong seasonality in regional wind fields^88^, affecting circulation and water mass properties down to ~ 1,000 m depth^89^ (Fig. 3c): North-easterly winds, typical in spring-summer, lead to the upwelling of Eastern North Atlantic Central Water (ENACW) bringing cold, nutrient rich waters with low pH and DO over the shelf^90,91^ (Fig. 3c). On the other hand, south-westerly winds, typical in autumn-winter, cause downwelling conditions that couple with the intrusion of warmer, oxygen-poor central waters in the shelf and slope^92–94^, contributing to the ventilation of bottom depths^95^. The upwelling/downwelling conditions exhibit intermittent character and vary over periods of days^96^. At sub-daily timescales, the notable strength of the semidiurnal tide in the region causes thermal fluctuations of up to 1.5 °C^97^. Based on historical records^98–102^, the overall conditions at the depths where corals were collected (150 m) range from 11.7 to 13.0 °C for temperature, 7.94 and 8.12 pH_T_ units (in situ, total scale), and 6.47 to 7.98 mL L^−1^ for DO.

### Sample collection and maintenance

Colonies of *Dendrophyllia cornigera* were collected as trammel net bycatch at 100–150 m depth (43° 23’ 55.8” N, 008° 42’ 2.9” W, Fig. 3a) in September 2019. Onboard, corals were placed in a thermal-insulated tank^38^ with seawater (~ 14 °C), and transported (~ 8 h after collection) to the aquaria facilities at the *Aquarium Finisterrae* (A Coruña, Spain). Upon arrival, corals were transferred to a 3,000 L flow-through maintenance tank with a constant flow of seawater pumped from 17 m depth, that was previously filtered to 40 μm and UV-treated (temperature: ~14 °C). Due to the COVID-19 pandemic, the corals remained in the husbandry tanks for 18 months. Corals were fed five times per week with a mixed diet of minced mussels, shrimp, hake and diluted algae paste (*Nannochloropsis oculata*, Phytobloom, Necton).

In order to obtain coral nubbins (coral fragments with 1–3 polyps) for the experiment, apical fragments from 26 coral colonies were cut with a rotary tool (Proxxon Micromot). Nubbins were glued to labelled methacrylate bases using cyanoacrylate glue (Loctite Super Glue 3) and placed into two 60 L flow-through tanks (26 L h^−1^, same water supply as in the maintenance tank) to facilitate their manipulation (Supplementary Fig. S6). Each tank had a submersible pump (Sicce Syncra Nano) for water mixing and remained in darkness. The initial weight of each nubbin was assessed before and after glueing it to the methacrylate base following the buoyant weight technique^103,104^ (see Sect. ❝Coral skeletal growth❞). Coral nubbins were fed five times per week with freshly hatched *Artemia salina* nauplii at concentrations similar to those used in previous studies (~ 1.25 nauplii mL^−1^^69^). This feeding ensured homogeneous food distribution across the polyps while facilitating subsequent cleaning, making it the most efficient choice given the long-term duration of the experiment.

### Experimental design and setup

**Fig. 4:**
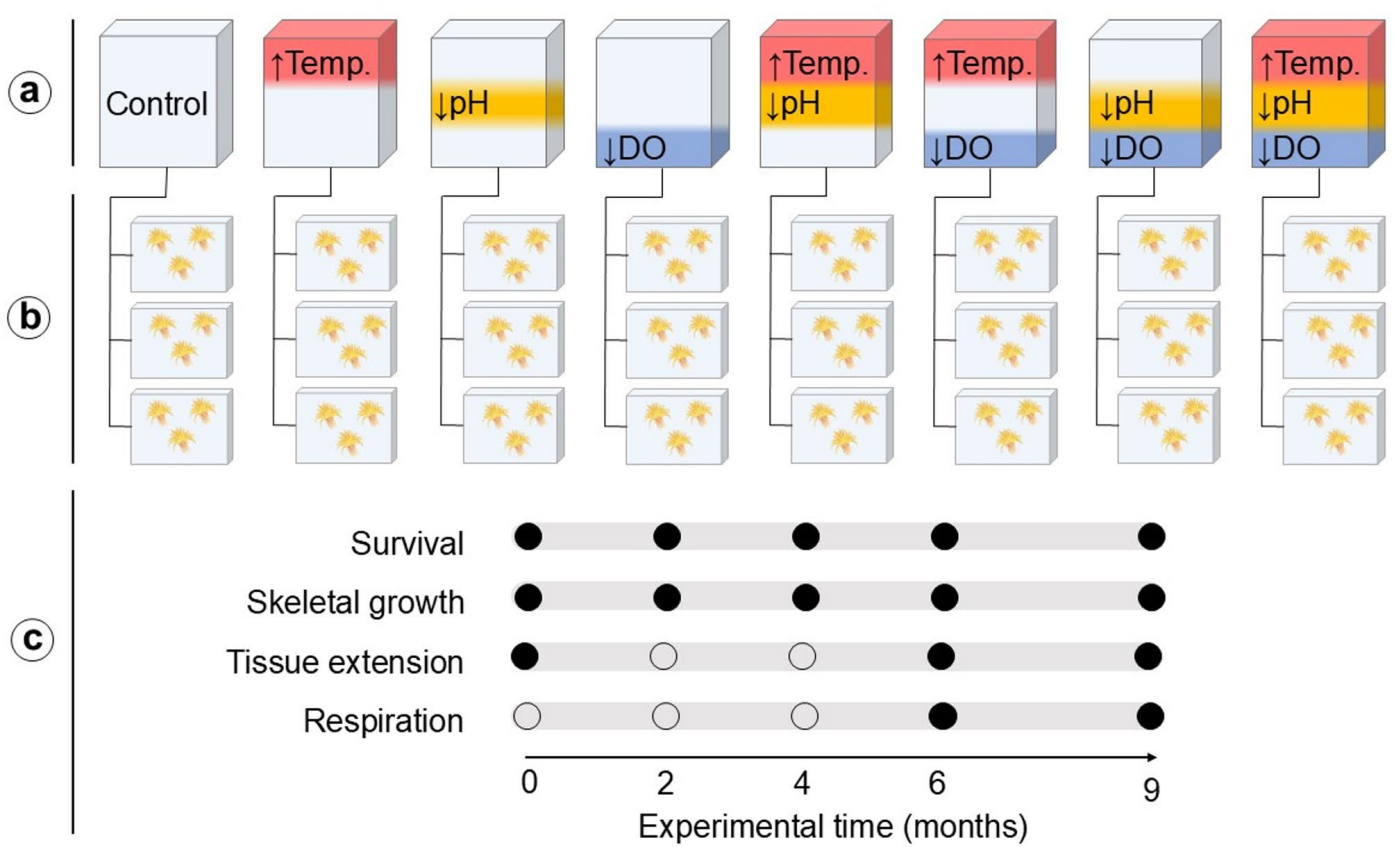
Experimental design and measured response variables across the experiment. (**a**) Treatments based on the current in situ conditions (white, control) and the IPCC SSP5-8.5 scenario for temperature (12 vs. 15 °C, red), pH_T_ (~ 7.99 vs. 7.69, total scale at in situ temperature, yellow) and Dissolved Oxygen (DO) concentration (~ 6.4 vs. 4.7 mL L^−1^, blue). Treatments are depicted from left to right: Control, elevated temperature, low pH, low oxygen, combined elevated temperature and low pH, combined elevated temperature and low oxygen, combined low pH and low oxygen, and combined elevated temperature, low pH and low oxygen. Water parameters for each treatment were modified in their corresponding header tank that supplied to (**b**) three replicated experimental aquaria, holding three coral nubbins each (**c**) Measured response variables and time of the analyses over the duration of the experiment: filled circles represent conducted analyses on a specific response variable at the corresponding experimental time (0, 2, 4, 6 and 9 months); blank circles indicate periods when analyses for that response variable were not conducted.

To assess the effects of elevated temperature, low pH and low oxygen, as well as their potential interactions, a full factorial design was set based on two scenarios: (1) current in situ conditions (control) based on field studies (see Sect. ❝Experimental conditions❞), and (2) projected end-of-the-century conditions for the study area under the RCP 8.5 scenario^9,105^ with three environmental parameters as factors: temperature (12 vs. 15 °C), pH_T_ (ambient vs. 7.69, equivalent to 1,000 ppm *p*CO_2_) and DO (ambient vs. 4.7 mL L^−1^). Ambient pH_T_ and DO (~ 7.99 pH_T_ and 6.4 mL L^−1^, respectively) in the experimental aquaria were established as current in situ values for the study area. The resulting eight treatments were adjusted in 80 L polycarbonate header tanks, which were supplied with natural seawater pumped from 17 m depth and treated through a flow-through system (~ 500 L h^−1^ inflow rate) (Fig. 4a). Seawater was chilled to 9 °C by an industrial chiller (Kosner KMCi-10), filtered to 5 μm and treated with UV light (Wedeco Rex) to prevent bacterial growth. Each header tank continuously supplied seawater (25 L h^−1^) to three 5 L experimental aquaria (Fig. 4b, Supplementary Fig. S7). A submersible pump (Sicce Syncra Nano) in each aquarium ensured constant water circulation. To achieve and maintain the target conditions for each treatment, two sets of microcomputers (Raspberry Pi 3) continuously monitored and manipulated temperature (°C), pH (pH units, NBS scale) and DO (% air saturation) in the eight header tanks to the targeted experimental values. For that purpose, a set of temperature (DS18B20), pH and DO (both Atlas Scientific Lab Grade) probes were set in each header tank and connected to the controllers. The temperature was increased using submersible heaters (Marina 300 W) activated by the controllers and maintained using 60 L water baths, each hosting experimental aquaria from two different treatments with the same target temperature^46^. The pH was lowered by injecting pure CO_2_ gas in the header tanks, using solenoid valves connected to the controllers and regulating the circulation of gas from a 13.4 L gas cylinder. DO was lowered by injecting pure N_2_ gas, following the same setup as for CO_2_, and using a 50 L pure N_2_ gas cylinder. Additional details on the aquaria setup are available in Gutiérrez-Zárate et al.^46^.

A total of 72 *D. cornigera* nubbins were randomly assigned to the different experimental aquaria using a random number generator, holding three nubbins each (9 per treatment). This distribution was later checked to ensure that all treatments included nubbins from multiple colonies while nubbin weight was not significantly different among the experimental aquaria (2.5 ± 0.5 g, mean ± standard deviation, SD; Kruskal-Wallis test, *p* = 0.22).

To acclimate the nubbins to the targeted treatment values, water parameters inside the experimental aquaria were gradually adjusted (ramping) as follows: for temperature, a daily change of 0.5 °C over 5 days^43,44,69^; for pH, a daily decrease of 0.03 pH units over 11 days^20^; and for DO, a daily decrease of 5% air saturation over 6 days (being the minimum substantial difference that could be achieved by the probes). After 15 d, all target experimental values were achieved. Corals were fed five times per week with freshly hatched *A. salina* nauplii (~ 1.25 nauplii mL^−1^) and maintained in the dark for 9 months.

All the water parameters were periodically monitored and compared with the target values to ensure the accuracy of the system. Temperature and DO were measured daily (YSI ProODO), salinity was assessed weekly (WTW 350i multiparameter device equipped with a ConOx probe) and pH was measured by spectrophotometry^106^ every 1–2 months. Total alkalinity from the aquaria system was measured using the double-ended potentiometric technique^107,108^ every 1–2 months. Dissolved inorganic nutrient concentrations (nitrite, nitrate, silicate and phosphate) from each experimental aquarium were measured after one, six and nine months using colorimetric methods^109,110^. Total alkalinity and pH were used to calculate additional carbonate system parameters at in situ conditions using the *carb* function of the R package *seacarb*^111^, considering the temperature, salinity, and silicate and phosphate concentrations. Finally, prokaryotic abundance in all experimental aquaria was quantified monthly using a CytoFLEX S flow cytometer^112^.

### Coral survival

Survival was assessed by counting the number of alive polyps per nubbin after 2, 4, 6 and 9 months during the experiment (Fig. 4c). Survival is reported as % of alive polyps per nubbin at each time point.

### Coral skeletal growth

The weight of the coral nubbins was assessed at the start of the experiment and after 2, 4, 6 and 9 months (Fig. 4c) using the buoyant weight technique^103,104^. Coral net weight in water was calculated by means of an analytical balance (OHAUS AX124, precision 0.1 mg) placed above a 5 L tank filled with seawater of the respective treatment. Temperature and salinity inside the 5 L tank were constantly monitored (using a ConOx probe connected to a WTW 350i multiparameter device) and used to calculate water density. The weight in air was calculated considering the coral net weight in water, the water density and the skeletal density of *D. cornigera* (2.63 g cm^−3^^20^). Feeding was stopped 48 h before weight measurements to prevent the potential influence of non-digested food^113^. Skeletal growth rates were calculated as the slope of the linear regression between the logarithmically transformed weight values and the experimental time and expressed as % d^−1^^67^.

### Coral tissue cover

Tissue surface area was assessed using photogrammetric analyses on 3D reconstructions of each nubbin at the beginning of the experiment, and after 6 and 9 months (Fig. 4c). To perform the 3D reconstructions, each nubbin was placed inside an 18 L aquarium filled with seawater of the corresponding treatment. A 3D-printed base was used to hold the nubbin and was attached to a rotating ring. A rotating motor below the aquarium was connected to a second rotating ring and was programmed by an Arduino microcomputer to automatically turn 12 degrees until achieving a full turn. The two rings, inside and below the aquarium, were connected through neodymium magnets to simultaneously rotate and achieve the rotation of the nubbin to the programmed degrees. The Arduino was connected to the shutter of a camera (Canon EOS 7D) that captured a photo after each rotation, obtaining 30 photos of the nubbin per turn (Supplementary Fig. S8a). A detailed description of the photogrammetric setup can be found in Romo et al.^114^. Each set of 30 photos was used to build 3D models of the nubbin, using Agisoft Metashape Professional v. 2.0.2^115^ and following the methodology applied by Bilan et al.^116^. Tissue surface quantification (cm^2^) was performed using MeshLab^117^ (Supplementary Fig. S8b). We calculated the rate of change in tissue surface for each nubbin as the slope of the linear regression between the logarithmically transformed tissue surface area and the experimental time, and expressed as % d^−1^.

### Coral respiration

Coral respiration was assessed by means of closed-cell incubations after 6 and 9 months (Fig. 4c). To account for background respiration, an empty methacrylate base with a small amount of glue was added to each aquarium at the start of the experiment to be used as a control. Coral nubbins and controls were incubated in closed-cell individual plastic chambers (494 mL in volume) filled with seawater from the respective treatment, air bubbles were removed and chambers were hermetically closed and maintained in the dark at the corresponding target temperature in a water bath (to ensure constant temperature during the incubation). Constant water movement inside the chambers was ensured by a Teflon-coated magnetic stirrer (CimarecTM, ThermoScientific). Respiration rates were calculated by measuring the DO (mg L^−1^) of the seawater inside the chambers using an optical oxygen sensor (YSI ProODO) at the beginning and at the end of the incubation time (24 h incubation). Respiration rates were normalised to the tissue surface area of each nubbin at the time of the measurement (µmol O_2_ cm^−2^ d^−1^).

### Statistical analyses

Linear mixed-effect models (LMMs, *lme4* package^118^) were used for hypothesis testing and to account for data dependency (several nubbins per experimental aquarium and several individuals from the same colony distributed in different treatments). Polyp number of each nubbin was excluded as covariate from the models after visual exploration of the data (Supplementary Fig. S9). To analyse skeletal growth and tissue cover data, treatment was used as a fixed effect, whereas the aquarium and colony were used as random effects. For respiration data, time and its interaction with treatment were included as fixed effects, and coral IDs were nested in aquarium and colony to account for repeated measurements. Model diagnostics were assessed using the *lmtest*^119^ and *performance*^120^ packages. Post-hoc comparisons were performed using the *emmeans* package^121^. To assess the output of the statistical models, graphic model predictions displaying the means and 95% confidence interval (CI) were plotted using the *ggpredict* function from the *ggeffects* package^122^. All statistical analyses and graphical presentations of the response variables were performed using R v. 4.3.1^123^ and the *ggplot* package^124^.

## Supplementary Information

Below is the link to the electronic supplementary material.

Supplementary Material 1

## Data Availability

Data generated and analysed in this study are available in the data repository PANGAEA. The datasets include: coral skeletal growth rates for the experimental time 10.1594/PANGAEA.966744^125^ and for each measuring time 10.1594/PANGAEA.978153^126^; respiration rates, 10.1594/PANGAEA.966748^127^; and tissue cover rates, 10.1594/PANGAEA.972862^128^.

## References

[1] Jones, C. G., Lawton, J. H. & Shachak, M. Organisms as ecosystem engineers. *Oikos* **69**, 373–386 (1994).

[2] Bourque, J. R. & Demopoulos, A. W. J. The influence of different deep-sea coral habitats on sediment macrofaunal community structure and function. *PeerJ* **6**, e5276 (2018). 30042896 10.7717/peerj.5276 PMC6055693

[3] Buhl-Mortensen, L. et al. Biological structures as a source of habitat heterogeneity and biodiversity on the deep ocean margins. *Mar. Ecol.* **31**, 21–50 (2010).

[4] Demopoulos, A. W. J., Bourque, J. R. & Frometa, J. Biodiversity and community composition of sediment macrofauna associated with deep-sea *Lophelia pertusa* habitats in the Gulf of Mexico. *Deep Sea Res. Part I* **93**, 91–103 (2014).

[5] Rueda, J. L. et al. in *In Mediterranean Cold-Water Corals: Past, Present and Future: Understanding the Deep-Sea Realms of Coral*. 295–333 (eds Orejas, C. & Jiménez, C.) (Springer International Publishing, 2019). 10.1007/978-3-319-91608-8_29

[6] Levin, L. A. Le Bris, N. The deep ocean under climate change. *Science* **350**, 766–768 (2015). 26564845 10.1126/science.aad0126

[7] Morato, T. et al. Climate-induced changes in the suitable habitat of cold-water corals and commercially important deep-sea fishes in the North Atlantic. *Glob. Change Biol.* **26**, 2181–2202 (2020). 10.1111/gcb.14996 PMC7154791 32077217

[8] Brito-Morales, I. et al. Climate velocity reveals increasing exposure of deep-ocean biodiversity to future warming. *Nat. Clim. Chang.* **10**, 576–581 (2020).

[9] Sweetman, A. K. et al. Major impacts of climate change on deep-sea benthic ecosystems. *Elem. Sci. Anth*. **5**, 4 (2017).

[10] Davies, A. J., Wisshak, M., Orr, J. C. & Murray Roberts, J. Predicting suitable habitat for the cold-water coral *Lophelia pertusa* (Scleractinia). *Deep Sea Res. Part I*. **55**, 1048–1062 (2008).

[11] Davies, A. J. & Guinotte, J. M. Global habitat suitability for Framework-Forming Cold-Water corals. *PLOS ONE*. **6**, e18483 (2011). 21525990 10.1371/journal.pone.0018483 PMC3078123

[12] Beck, K. K. et al. Ontogenetic differences in the response of the cold-water coral *Caryophyllia huinayensis* to ocean acidification, warming and food availability. *Sci. Total Environ.* **900**, 165565 (2023). 37495133 10.1016/j.scitotenv.2023.165565

[13] Brooke, S., Ross, S. W., Bane, J. M., Seim, H. E. & Young, C. M. Temperature tolerance of the deep-sea coral *Lophelia pertusa* from the southeastern united States. *Deep Sea Res. Part II*. **92**, 240–248 (2013).

[14] Lunden, J. J., McNicholl, C. G., Sears, C. R., Morrison, C. L. & Cordes, E. E. Acute survivorship of the deep-sea coral *Lophelia pertusa* from the Gulf of Mexico under acidification, warming, and deoxygenation. *Front Mar. Sci* **1**, 78 (2014).

[15] Riebesell, U., Fabry, V. J., Hansson, L. & Gattuso, J. P. *Guide To Best Practices for Ocean Acidification Research and Data Reporting* (Office for Official Publications of the European Communities, 2011).

[16] Tanhua, T., Körtzinger, A., Friis, K., Waugh, D. W. & Wallace, D. W. R. An estimate of anthropogenic CO_2_ inventory from decadal changes in oceanic carbon content. *Proceedings of the National Academy of Sciences* 104, 3037–3042 (2007). 10.1073/pnas.0606574104 PMC1802019 17360605

[17] Büscher, J. V., Form, A. U. & Riebesell, U. Interactive effects of ocean acidification and warming on Growth, fitness and survival of the Cold-Water coral *Lophelia pertusa* under different food availabilities. *Front Mar. Sci* **4**, 101 (2017).

[18] Büscher, J. V., Form, A. U., Wisshak, M., Kiko, R. & Riebesell, U. Cold-water coral ecosystems under future ocean change: live coral performance vs. framework dissolution and bioerosion. *Limnol. Oceanogr.* **67**, 2497–2515 (2022).

[19] Martínez-Dios, A. et al. Effects of low pH and feeding on calcification rates of the cold-water coral *Desmophyllum dianthus*. *PeerJ* **8**, e8236 (2020). 31915573 10.7717/peerj.8236 PMC6942680

[20] Movilla, J. et al. Differential response of two mediterranean cold-water coral species to ocean acidification. *Coral Reefs*. **33**, 675–686 (2014).

[21] Hennige, S. J. et al. Crumbling reefs and Cold-Water coral habitat loss in a future ocean: evidence of coralporosis as an indicator of habitat integrity. *Front Mar. Sci* **7**, 668 (2020).

[22] Barnhill, K. A. et al. Incorporating dead material in ecosystem assessments and projections. *Nat. Clim. Chang.* **13**, 113–115 (2023).

[23] Keeling, R. F., Körtzinger, A. & Gruber, N. Ocean deoxygenation in a warming world. *Annual Rev. Mar. Sci.* **2**, 199–229 (2010). 10.1146/annurev.marine.010908.163855 21141663

[24] Mora, C. et al. Biotic and human vulnerability to projected changes in ocean biogeochemistry over the 21st century. *PLoS Biol.* **11**, e1001682 (2013). 24143135 10.1371/journal.pbio.1001682 PMC3797030

[25] Georgian, S. E. et al. Oceanographic patterns and carbonate chemistry in the vicinity of cold-water coral reefs in the Gulf of mexico: implications for resilience in a changing ocean. *Limnol. Oceanogr.* **61**, 648–665 (2016).

[26] Hanz, U. et al. Environmental factors influencing benthic communities in the oxygen minimum zones on the Angolan and Namibian margins. *Biogeosciences* **16**, 4337–4356 (2019).

[27] Hebbeln, D. et al. Cold-water coral reefs thriving under hypoxia. *Coral Reefs*. 10.1007/s00338-020-01934-6 (2020).

[28] Orejas, C. et al. *Madrepora oculata* forms large frameworks in hypoxic waters off Angola (SE Atlantic). *Sci. Rep.* **11**, 15170 (2021). 34312452 10.1038/s41598-021-94579-6 PMC8313707

[29] Ramos, A., Sanz, J. L., Ramil, F., Agudo, L. M. & Presas-Navarro, C. in *In Deep-Sea Ecosystems Off Mauritania: Research of Marine Biodiversity and Habitats in the Northwest African Margin*. 481–525 (eds Ramos, A., Ramil, F. & Sanz, J. L.) (Springer Netherlands, 2017). 10.1007/978-94-024-1023-5_13

[30] Dodds, L. A., Roberts, J. M., Taylor, A. C. & Marubini, F. Metabolic tolerance of the cold-water coral *Lophelia pertusa* (Scleractinia) to temperature and dissolved oxygen change. *J. Exp. Mar. Biol. Ecol.* **349**, 205–214 (2007).

[31] Gori, A. et al. Natural hypoxic conditions do not affect the respiration rates of the cold-water coral *Desmophyllum pertusum* (*Lophelia pertusa*) living in the Angola margin (Southeastern Atlantic Ocean). *Deep Sea Res. Part I*. **197**, 104052 (2023).

[32] Ban, S. S., Graham, N. A. J. & Connolly, S. R. Evidence for multiple stressor interactions and effects on coral reefs. *Glob. Change Biol.* **20**, 681–697 (2014). 10.1111/gcb.12453 24166756

[33] Gao, K., Gao, G., Wang, Y. & Dupont, S. Impacts of ocean acidification under multiple stressors on typical organisms and ecological processes. *Mar. Life Sci. Technol.* **2**, 279–291 (2020).

[34] Pörtner, H. O. Integrating climate-related stressor effects on marine organisms: unifying principles linking molecule to ecosystem-level changes. *Mar. Ecol. Prog. Ser.* **470**, 273–290 (2012).

[35] Pendleton, L. H., Hoegh-Guldberg, O., Langdon, C. & Comte, A. Multiple stressors and ecological complexity require a new approach to coral reef research. *Front Mar. Sci* **3**, 36 (2016).

[36] Gori, A. et al. Physiological response of the cold-water coral *Desmophyllum dianthus* to thermal stress and ocean acidification. *PeerJ* **4**, e1606 (2016). 26855864 10.7717/peerj.1606 PMC4741066

[37] Hennige, S. J. et al. Hidden impacts of ocean acidification to live and dead coral framework. *Proceedings of the Royal Society B: Biological Sciences* 282, 20150990 (2015). 10.1098/rspb.2015.0990 PMC4632617 26290073

[38] Orejas, C. et al. in *In Mediterranean Cold-Water Corals: Past, Present and Future: Understanding the Deep-Sea Realms of Coral*. 435–471 (eds Orejas, C. & Jiménez, C.) (Springer International Publishing, 2019). 10.1007/978-3-319-91608-8_38

[39] Reynaud, S. et al. Dendrophylliidae cold-water corals in a warm ocean: the effect of exposure duration on their physiological response. *Deep Sea Res. Part II*. **193**, 104962 (2021).

[40] Movilla, J. et al. Resistance of two mediterranean Cold-Water Coral species to low-pH conditions. *Water* **6**, 59–67 (2014).

[41] Carreiro-Silva, M. et al. Molecular mechanisms underlying the physiological responses of the cold-water coral *Desmophyllum dianthus* to ocean acidification. *Coral Reefs*. **33**, 465–476 (2014).

[42] Kurman, M. D., Gómez, C. E., Georgian, S. E., Lunden, J. J. & Cordes, E. E. Intra-Specific variation reveals potential for adaptation to ocean acidification in a Cold-Water coral from the Gulf of Mexico. *Front. Mar. Sci.* **4**, 111 (2017).

[43] Gori, A., Reynaud, S., Orejas, C., Gili, J. M. & Ferrier-Pagès, C. Physiological performance of the cold-water coral *Dendrophyllia cornigera* reveals its preference for temperate environments. *Coral Reefs*. **33**, 665–674 (2014).

[44] Naumann, M. S. & Orejas, C. Ferrier-Pagès, C. High thermal tolerance of two mediterranean cold-water coral species maintained in Aquaria. *Coral Reefs*. **32**, 749–754 (2013).

[45] Rodolfo-Metalpa, R. et al. Calcification is not the achilles’ heel of cold-water corals in an acidifying ocean. *Glob. Change Biol.* **21**, 2238–2248 (2015). 10.1111/gcb.12867 25641230

[46] Gutiérrez-Zárate, C. et al. An Aquaria set-up for long-term, multiple-stressor research in marine organisms. *Methods Ecol. Evol.* **16**, 414–426 (2025).

[47] Castellan, G., Angeletti, L., Taviani, M. & Montagna, P. The yellow coral *Dendrophyllia cornigera* in a warming ocean. *Front Mar. Sci* **6**, 692 (2019).

[48] Zibrowius, H. *Les scléractiniaires De La Méditerranée Et De l’Atlantique nord-oriental* (Institut océanographique, 1980).

[49] Barton, E. D. et al. The transition zone of the Canary current upwelling region. *Prog. Oceanogr.* **41**, 455–504 (1998).

[50] Brito, A. & Ocaña, B. *Corals of the Canary Islands: Skeleton Anthozoa of the Littoral and Deep Bottoms* (Francisco Lemus, 2004).

[51] Addamo, A. M. et al. Merging scleractinian genera: the overwhelming genetic similarity between solitary *Desmophyllum* and colonial *Lophelia*. *BMC Evol. Biol.* **16**, 108 (2016). 27193263 10.1186/s12862-016-0654-8 PMC4870751

[52] Chapron, L. et al. Resilience of cold-water coral holobionts to thermal stress. *Proc. Royal Soc. B: Biol. Sci.* **288**, 20212117 (2021). 10.1098/rspb.2021.2117 PMC8670956 34905712

[53] Chemel, M. et al. Cold-water coral mortality under ocean warming is associated with pathogenic bacteria. *Environ. Microbiome*. **19**, 76 (2024). 39407340 10.1186/s40793-024-00622-0 PMC11481251

[54] Vertino, A., Stolarski, J., Bosellini, F. R. & Taviani, M. in *In the Mediterranean Sea: its History and Present Challenges*. 257–274 (eds Goffredo, S. & Dubinsky, Z.) (Springer Netherlands, 2014). 10.1007/978-94-007-6704-1_14

[55] Vertino, A., Taviani, M. & Corselli, C. in *In Mediterranean Cold-Water Corals: Past, Present and Future: Understanding the Deep-Sea Realms of Coral*. 67–83 (eds Orejas, C. & Jiménez, C.) (Springer International Publishing, 2019). 10.1007/978-3-319-91608-8_9

[56] Athanasiou, M. et al. Sea surface temperatures and environmental conditions during the warm pliocene interval (~ 4.1–3.2 Ma) in the Eastern mediterranean (Cyprus). *Glob. Planet Change*. **150**, 46–57 (2017).

[57] Herbert, T. D., Ng, G. & Cleaveland Peterson, L. Evolution of mediterranean sea surface temperatures 3.5–1.5 Ma: regional and hemispheric influences. *Earth Planet. Sci. Lett.* **409**, 307–318 (2015).

[58] Wienberg, C. et al. Scleractinian cold-water corals in the Gulf of Cádiz—First clues about their Spatial and Temporal distribution. *Deep Sea Res. Part I*. **56**, 1873–1893 (2009).

[59] Maier, C. et al. Effects of elevated *p*CO_2_ and feeding on net calcification and energy budget of the mediterranean cold-water coral *Madrepora oculata*. *J. Exp. Biol.* **219**, 3208–3217 (2016). 27471280 10.1242/jeb.127159

[60] Freiwald, A. et al. The white coral community in the central mediterranean sea revealed by ROV surveys. *Oceanography* **22**, 58–74 (2009).

[61] Vaquer-Sunyer, R. & Duarte, C. M. Thresholds of hypoxia for marine biodiversity. *Proceedings of the National Academy of Sciences* 105, 15452–15457 (2008). 10.1073/pnas.0803833105 PMC2556360 18824689

[62] Johnson, M. D., Swaminathan, S. D., Nixon, E. N., Paul, V. J. & Altieri, A. H. Differential susceptibility of reef-building corals to deoxygenation reveals remarkable hypoxia tolerance. *Sci. Rep.* **11**, 23168 (2021). 34848743 10.1038/s41598-021-01078-9 PMC8632909

[63] Da Ros, Z. et al. Food preferences of mediterranean Cold-Water corals in captivity. *Front Mar. Sci* **9**, 867656 (2022).

[64] Gori, A., Grover, R., Orejas, C., Sikorski, S. & Ferrier-Pagès, C. Uptake of dissolved free amino acids by four cold-water coral species from the mediterranean sea. *Deep Sea Res. Part II*. **99**, 42–50 (2014).

[65] Gori, A. et al. Biochemical composition of the cold-water coral *Dendrophyllia cornigera* under contrasting productivity regimes: insights from lipid biomarkers and compound-specific isotopes. *Deep Sea Res. Part I*. **141**, 106–117 (2018).

[66] Boavida, J., Becheler, R., Addamo, A. M., Sylvestre, F. & Arnaud-Haond, S. in *In Mediterranean Cold-Water Corals: Past, Present and Future: Understanding the Deep-Sea Realms of Coral*. 357–372 (eds Orejas, C. & Jiménez, C.) (Springer International Publishing, 2019). 10.1007/978-3-319-91608-8_31

[67] Orejas, C. et al. Long-term growth rates of four mediterranean cold-water coral species maintained in aquaria. *Mar. Ecol. Prog. Ser.* **429**, 57–65 (2011).

[68] Naumann, M. S., Orejas, C. & Wild, C. Ferrier-Pagès, C. First evidence for zooplankton feeding sustaining key physiological processes in a scleractinian cold-water coral. *J. Exp. Biol.* **214**, 3570–3576 (2011). 21993785 10.1242/jeb.061390

[69] Gori, A., Reynaud, S., Orejas, C. & Ferrier-Pagès, C. The influence of flow velocity and temperature on zooplankton capture rates by the cold-water coral *Dendrophyllia cornigera*. *J. Exp. Mar. Biol. Ecol.* **466**, 92–97 (2015).

[70] Tsounis, G. et al. Prey-capture rates in four mediterranean cold water corals. *Mar. Ecol. Prog. Ser.* **398**, 149–155 (2010).

[71] Edmunds, P. J. Zooplanktivory ameliorates the effects of ocean acidification on the reef coral *Porites* spp. *Limnol. Oceanogr.* **56**, 2402–2410 (2011).

[72] Holcomb, M., McCorkle, D. C. & Cohen, A. L. Long-term effects of nutrient and CO_2_ enrichment on the temperate coral *Astrangia poculata* (Ellis and Solander, 1786). *J. Exp. Mar. Biol. Ecol.* **386**, 27–33 (2010).

[73] Georgian, S. E. et al. Biogeographic variability in the physiological response of the cold-water coral *Lophelia pertusa* to ocean acidification. *Mar. Ecol.* **37**, 1345–1359 (2016).

[74] Büscher, J. V. et al. Water mass characteristics and hydrodynamics at an inshore versus an offshore mid-Norwegian cold-water coral reef habitat. *Front Mar. Sci* **11**, 1363542 (2024).

[75] Findlay, H. S. et al. Tidal downwelling and implications for the carbon biogeochemistry of cold-water corals in relation to future ocean acidification and warming. *Glob. Change Biol.* **19**, 2708–2719 (2013). 10.1111/gcb.12256 23666812

[76] Juva, K., Kutti, T., Chierici, M., Dullo, W. C. & Flögel, S. Cold-Water coral reefs in the langenuen Fjord, Southwestern Norway—A window into future environmental change. *Oceans* **2**, 583–610 (2021).

[77] Carrick, J. V., Mienis, F., Cordes, E. E., Demopoulos, A. W. J. & Davies, A. J. Gulf Stream intrusion and deep current upwelling drive dynamic patterns of temperature and food supply within cold-water coral reefs. *Limnol. Oceanogr.* **69**, 2193–2210 (2024).

[78] Mienis, F. et al. Cold-water coral growth under extreme environmental conditions, the Cape Lookout area, NW Atlantic. *Biogeosciences* **11**, 2543–2560 (2014).

[79] Cabrerizo, M. J. & Marañón, E. Net effect of environmental fluctuations in multiple global-change drivers across the tree of life. *Proceedings of the National Academy of Sciences* 119, e2205495119 (2022). 10.1073/pnas.2205495119 PMC9371701 35914141

[80] Rivest, E. B., Comeau, S. & Cornwall, C. E. The role of natural variability in shaping the response of coral reef organisms to climate change. *Curr. Clim. Change Rep.* **3**, 271–281 (2017).

[81] Tanvet, C. et al. Corals adapted to extreme and fluctuating seawater pH increase calcification rates and have unique symbiont communities. *Ecol. Evol.* **13**, e10099 (2023). 37261315 10.1002/ece3.10099 PMC10227177

[82] Wall, C. B. et al. The effects of environmental history and thermal stress on coral physiology and immunity. *Mar. Biol.* **165**, 56 (2018).

[83] Waller, R. G., Goode, S., Tracey, D., Johnstone, J. & Mercier, A. A review of current knowledge on reproductive and larval processes of deep-sea corals. *Mar. Biol.* **170**, 58 (2023).

[84] Rakka, M., Carreiro-Silva, M. & Larsson, A. I. Adverse effects of ocean acidification on embryonic survival of the cold-water coral *Desmophyllum pertusum*. *Invertebr. Biol.* **144**, 1 (2025).

[85] IPCC. Climate Change 2023: Synthesis Report. Contribution of Working Groups I, II and III to the Sixth Assessment Report of the Intergovernmental Panel on Climate Change. 10.59327/IPCC/AR6-9789291691647%3E (2023).

[86] Enrichetti, F. et al. Facies created by the yellow coral *Dendrophyllia cornigera* (Lamarck, 1816): Origin, substrate preferences and habitat complexity. *Deep Sea Res. Part I*. **195**, 104000 (2023).

[87] Wooster, W., Bakun, A. & McLain, D. The seasonal upwelling cycle along the Eastern boundary of the North Atlantic. *J. Mar. Res.* **34**, 2 (1976).

[88] Herrera, J. L., Rosón, G., Varela, R. A. & Piedracoba, S. Variability of the Western Galician upwelling system (NW Spain) during an intensively sampled annual cycle. An EOF analysis approach. *J. Mar. Syst.* **72**, 200–217 (2008).

[89] Prieto, E. et al. Seasonality of intermediate waters hydrography West of the Iberian Peninsula from an 8 year semiannual time series of an oceanographic section. *Ocean Sci.* **9**, 411–429 (2013).

[90] Borges, A. V. & Frankignoulle, M. Aspects of dissolved inorganic carbon dynamics in the upwelling system off the Galician Coast. *J. Mar. Syst.* **32**, 181–198 (2002).

[91] Ríos, A. F., Pérez, F. F. & Fraga, F. Water masses in the upper and middle North Atlantic ocean East of the Azores. *Deep Sea Res. Part. A*. **39**, 645–658 (1992).

[92] Álvarez-Salgado, X. A. et al. The Portugal coastal counter current off NW spain: new insights on its biogeochemical variability. *Prog. Oceanogr.* **56**, 281–321 (2003).

[93] Perez, F., Mouriño, C., Fraga, F. & Rios, A. Displacement of water masses and remineralization rates off the Iberian Peninsula by nutrient anomalies. *J. Mar. Res.* **51**, 4 (1993).

[94] Pingree, R. D. & Le Cann, B. Structure, strength and seasonality of the slope currents in the Bay of Biscay region. *J. Mar. Biol. Association United Kingd.* **70**, 857–885 (1990).

[95] Reboreda, R. et al. Oxygen in the Iberian margin: A modelling study. *Prog. Oceanogr.* **131**, 1–20 (2015).

[96] Fraga, F., Mouriño, C. & Manríquez, M. Las Masas de Agua En La Costa de galicia: junio-octubre. *Resultados Expediciones Científicas*. **10**, 51–77 (1982).

[97] García Lafuente, J. et al. Low-frequency variability of the mediterranean undercurrent off Galicia, Northwestern Iberian Peninsula. *J. Mar. Syst.* **74**, 351–363 (2008).

[98] Lange, N. et al. Synthesis product for ocean time series (SPOTS) – a ship-based biogeochemical pilot. *Earth Syst. Sci. Data*. **16**, 1901–1931 (2024).

[99] Lauvset, S. K. et al. The annual update GLODAPv2.2023: the global interior ocean biogeochemical data product. *Earth Syst. Sci. Data*. **16**, 2047–2072 (2024).

[100] Padin, X. A., Velo, A. & Pérez, F. F. ARIOS: a database for ocean acidification assessment in the Iberian upwelling system (1976–2018). *Earth Syst. Sci. Data*. **12**, 2647–2663 (2020).

[101] Prieto, E., González-Pola, C., Lavín, A. & Holliday, N. P. Interannual variability of the Northwestern Iberia deep ocean: response to large-scale North Atlantic forcing. *J. Geophys. Research: Oceans*. **120**, 832–847 (2015).

[102] Tel, E. et al. IEOOS: the Spanish Institute of oceanography observing system. *Ocean Sci.* **12**, 345–353 (2016).

[103] Jokiel, P., Maragos, J. E. & Franzisket, L. Coral growth: buoyant weight technique. in *Coral Reefs: Research Methods* 5 (eds Stoddart, D. R. & Johannes, R. E.) (1978).

[104] Davies, P. Short-term growth measurements of corals using an accurate buoyant weighing technique. *Mar. Biol.* **101**, 389–395 (1989).

[105] IPCC. Climate Change 2014: Synthesis Report. Contribution of Working Groups I, II and III to the Fifth Assessment Report of the Intergovernmental Panel on Climate Change. 10.7927/H4542KJV%3E (2014).

[106] Clayton, T. D. & Byrne, R. H. Spectrophotometric seawater pH measurements: total hydrogen ion concentration scale calibration of m-cresol purple and at-sea results. *Deep Sea Res. Part I*. **40**, 2115–2129 (1993).

[107] Pérez, F. F., Ríos, A. F., Rellán, T. & Álvarez, M. Improvements in a fast potentiometric seawater alkalinity determination. *Ciencias Marinas* **26**, 463–478 (2000).

[108] Perez, F. F. & Fraga, F. A precise and rapid analytical procedure for alkalinity determination. *Mar. Chem.* **21**, 169–182 (1987).

[109] Becker, S. et al. GO-SHIP Repeat Hydrography Nutrient Manual: The precise and accurate determination of dissolved inorganic nutrients in Seawater, using continuous flow analysis methods. *Front. Mar. Sci.* **7**, 581790 (2020).

[110] Hydes, D. et al. *GO-SHIP Repeat Hydrography Manual: A Collection of Expert Reports and guidelines. IOCCP Report No 14, ICPO Publication Series No. 134, Version 1 (ed* (UNESCO/IOC) IOCCP Report, 2010).

[111] Gattuso, J. P. et al. seacarb: Seawater Carbonate Chemistry. https://cran.r-project.org/web/packages/seacarb/index.html (2023).

[112] Gasol, J. M., Zweifel, U. L., Peters, F., Fuhrman, J. A. & Hagström, Å. Significance of size and nucleic acid content heterogeneity as measured by flow cytometry in natural planktonic bacteria. *Appl. Environ. Microbiol.* **65**, 4475–4483 (1999). 10508078 10.1128/aem.65.10.4475-4483.1999 PMC91596

[113] Hennige, S. J. et al. Short-term metabolic and growth responses of the cold-water coral *Lophelia pertusa* to ocean acidification. *Deep Sea Res. Part II*. **99**, 27–35 (2014).

[114] Romo, A. et al. A cost-effective, open-source laboratory system for 3D photogrammetric analysis of corals. *Deep Sea Res. Part II*. **223**, 105525 (2025).

[115] AgiSoft PhotoScan Professional. http://www.agisoft.com/downloads/installer/ (2023).

[116] Bilan, M. et al. Vulnerability of six cold-water corals to sediment resuspension from bottom trawling fishing. *Mar. Pollut. Bull.* **196**, 115423 (2023). 37862847 10.1016/j.marpolbul.2023.115423

[117] Cignoni, P. et al. Meshlab: an open-source mesh processing tool. In *Eurographics Italian Chapter Conference* 2008, 129–136 (2008).

[118] Bates, D., Mächler, M., Bolker, B. & Walker, S. Fitting linear Mixed-Effects models using lme4. *J. Stat. Softw.* **67**, 1–48 (2015).

[119] Zeileis, A. & Hothorn, T. Diagnostic checking in regression relationships. *R News*. **2**, 7–10 (2002).

[120] Lüdecke, D., Ben-Shachar, M. S., Patil, I., Waggoner, P. & Makowski, D. Performance: an R package for Assessment, comparison and testing of statistical models. *J. Open. Source Softw.* **6**, 3139 (2021).

[121] Lenth, R. V. *emmeans: Estimated Marginal Means, aka Least-Squares Means*. https://CRAN.R-project.org/package=emmeans (2023).

[122] Lüdecke, D. Tidy data frames of marginal effects from regression models. *J. Open. Source Softw.* **3**, 772 (2018).

[123] R Core Team. R: A Language and Environment for Statistical Computing. https://www.R-project.org/ (R Foundation for Statistical & Computing, 2023).

[124] Wickham, H. *ggplot2: Elegant Graphics for Data Analysis* (Springer, 2016).

[125] Gutiérrez-Zárate, C. et al. Skeletal growth rates of the cold-water coral *Dendrophyllia cornigera* from a long-term multi-stressor experiment. 10.1594/PANGAEA.966744 (2024).

[126] Gutiérrez-Zárate, C. et al. Skeletal growth rates of the cold-water coral *Dendrophyllia cornigera* for each time interval of a long-term multi-stressor experiment. 10.1594/PANGAEA.978153 (2025).

[127] Gutiérrez-Zárate, C. et al. Respiration rates of the cold-water coral *Dendrophyllia cornigera* from a long-term multi-stressor experiment. 10.1594/PANGAEA.966748 (2024).

[128] Gutiérrez-Zárate, C. et al. Tissue extension rates of the cold-water coral *Dendrophyllia cornigera* from a long-term multi-stressor experiment. 10.1594/PANGAEA.972862 (2024).

