# Supplementary material for: Resistance of the cold-water coral Dendrophyllia cornigera to single and combined global change stressors
Source: Sci Rep. 2025 Nov 17;15:40306. doi: 10.1038/s41598-025-24028-1 (PMC12623817; doi:10.1038/s41598-025-24028-1)
Supplement: Supplementary file 1 — Supplementary Material 1 [file 41598_2025_24028_MOESM1_ESM.pdf]

SUPPLEMENTARY MATERIAL

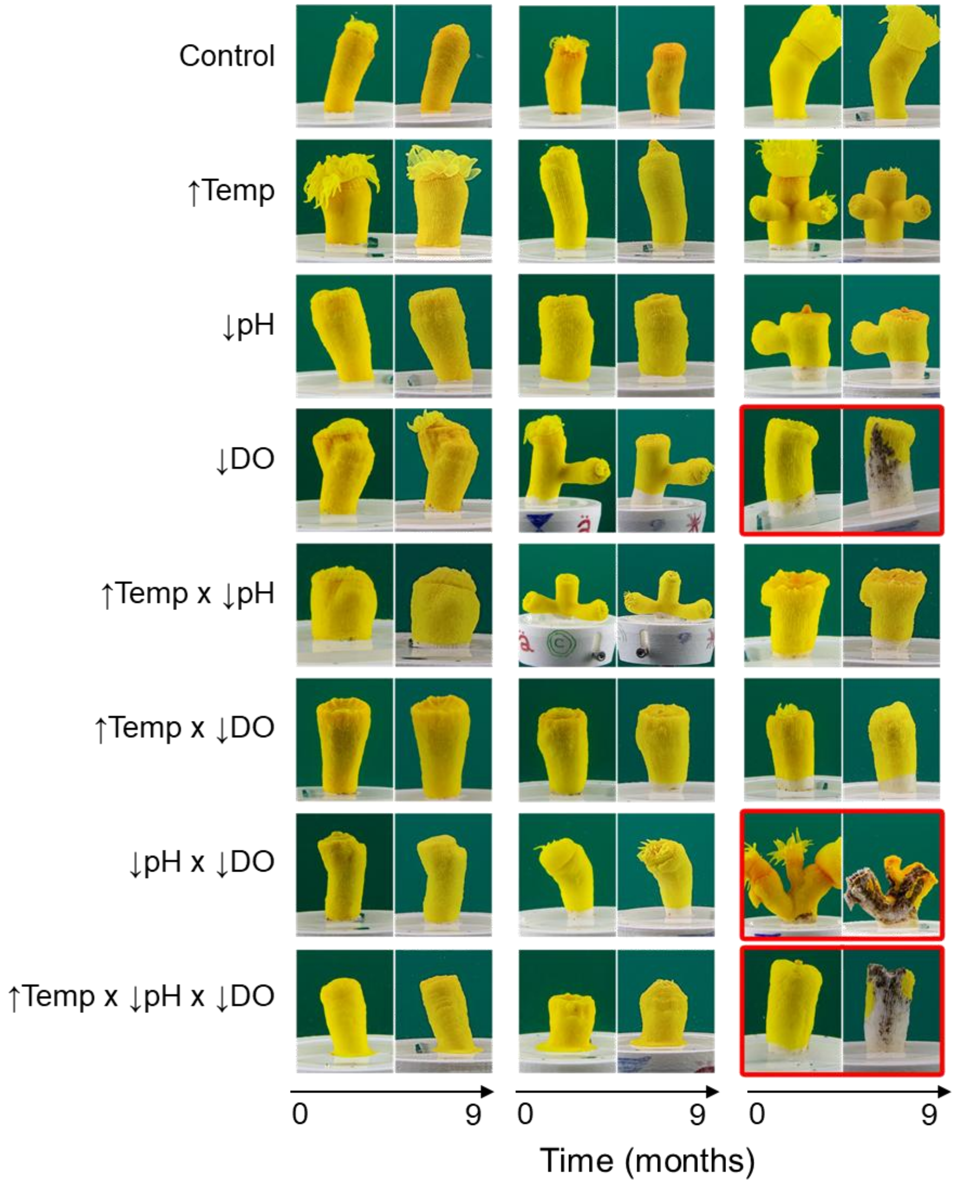

Supplementary Fig. S1. Representative images of *Dendrophyllia cornigera* nubbins at the beginning and after 9 months of experimental time under each treatment. Nubbins that experienced increased tissue necrosis and died are outlined in red. Treatments, from top to bottom, are: control, elevated temperature, low pH, low oxygen, combined elevated temperature and low pH, combined elevated temperature and low oxygen, combined low pH and low oxygen, and combined elevated temperature, low pH and low oxygen.

Supplementary Table S1. Number of polyps (total and per nubbin) and summary of response variables of *Dendrophyllia cornigera* for each treatment (mean ± SD). Survival was calculated per nubbin, as the percentage of alive polyps relative to its total number of polyps. Skeletal growth and tissue cover were calculated as the slope of the linear regression between the logarithmically transformed dry mass and tissue surface area, respectively, and the experimental time. Respiration after 6 and 9 months is normalised to tissue surface area and by dry mass.

Treatment	Total no. of polyps	Polyps per nubbin	Survival (%)	Skeletal growth (% d^{-1})	Tissue cover (% d^{-1})	Tissue Surface Area (cm^2)		Dry Mass (g)		Respiration by Tissue Area (µmol O_2 cm^{-2} d^{-1})		Respiration by Dry Mass (µmol O_2 g^{-1} d^{-1})	
						6 months	9 months	6 months	9 months	6 months	9 months	6 months	9 months
Control	19	2.1 ± 1.7	100.0 ± 0	0.033 ± 0.011	-0.010 ± 0.043	11.368 ± 4.232	11.616 ± 4.259	2.986 ± 1.615	2.986 ± 1.615	1.010 ± 0.232	0.801 ± 0.314	4.305 ± 1.661	3.402 ± 1.585
↑Temp	14	1.6 ± 0.7	100.0 ± 0	0.032 ± 0.027	0.006 ± 0.030	10.885 ± 2.752	11.236 ± 3.153	2.858 ± 1.068	2.858 ± 1.068	1.030 ± 0.333	1.010 ± 0.423	4.175 ± 1.505	4.166 ± 2.031
↓pH	12	1.3 ± 0.5	100.0 ± 0	0.030 ± 0.024	-0.014 ± 0.036	8.804 ± 1.650	8.985 ± 1.919	2.217 ± 1.165	2.337 ± 1.185	1.060 ± 0.326	0.626 ± 0.286	4.859 ± 2.099	3.375 ± 2.435
↓DO	14	1.6 ± 0.9	88.9 ± 33.1	0.026 ± 0.007	-0.014 ± 0.026	10.036 ± 4.594	9.911 ± 5.047	2.888 ± 1.648	2.888 ± 1.648	0.964 ± 0.273	0.683 ± 0.384	3.779 ± 1.080	2.547 ± 1.247
↑Temp x ↓pH	12	1.3 ± 0.7	100.0 ± 0	0.035 ± 0.024	-0.005 ± 0.029	8.635 ± 3.449	8.383 ± 4.086	2.041 ± 1.174	1.909 ± 1.167	1.110 ± 0.390	0.748 ± 0.244	6.219 ± 3.959	3.722 ± 1.993
↑Temp x ↓DO	13	1.4 ± 0.9	100.0 ± 0	0.023 ± 0.013	-0.016 ± 0.054	10.462 ± 4.630	10.653 ± 4.488	3.229 ± 2.335	3.129 ± 2.205	1.110 ± 0.249	0.715 ± 0.462	3.840 ± 1.337	2.587 ± 1.564
↓pH x ↓DO	15	1.5 ± 1.1	89.0 ± 33.3	0.023 ± 0.010	-0.012 ± 0.024	11.975 ± 6.068	11.577 ± 6.365	3.226 ± 2.197	3.094 ± 2.311	0.792 ± 0.242	0.850 ± 0.314	3.224 ± 0.985	3.449 ± 1.400
↑Temp x ↓pH x ↓DO	11	1.3 ± 0.5	88.9 ± 33.3	0.036 ± 0.026	0.016 ± 0.035	8.464 ± 3.973	9.097 ± 3.879	1.791 ± 0.865	1.761 ± 0.920	1.160 ± 0.217	1.030 ± 0.171	6.500 ± 2.634	5.809 ± 2.240

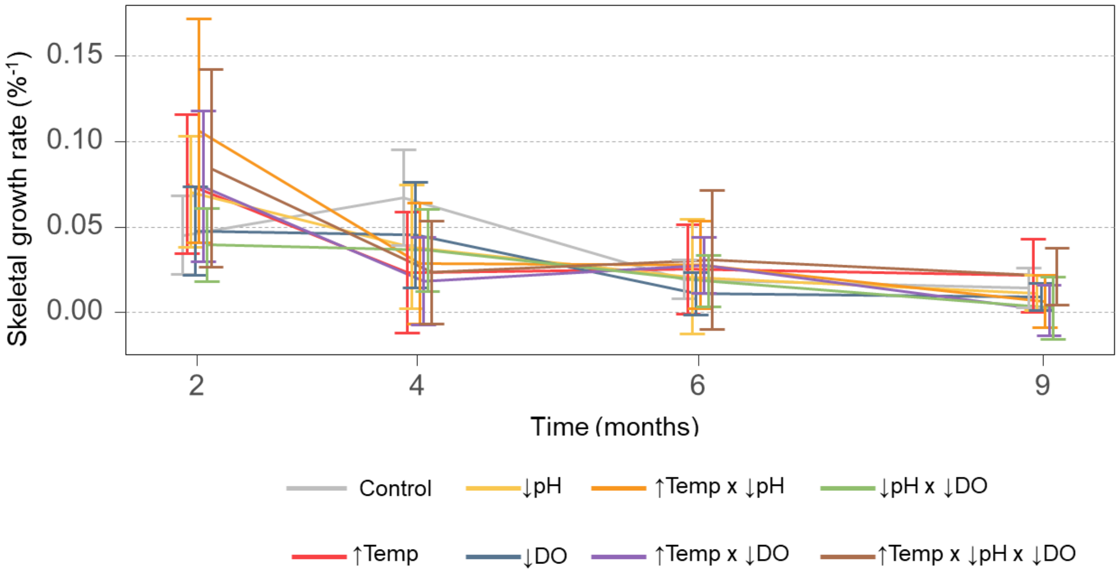

Supplementary Fig. S2. Skeletal growth rate of *Dendrophyllia cornigera* after 2, 4, 6, and 9 months of experimental time under the different treatments. Skeletal growth rates for each interval (mean ± SD) were calculated using the formula described in Orejas et al.[1]. Treatment conditions are: control (grey), elevated temperature (↑Temp, red), low pH (↓pH, yellow), low oxygen (↓DO, blue), combined elevated temperature and low pH (↑Temp x ↓pH, orange), combined elevated temperature and low oxygen (↑Temp x ↓DO, purple), combined low pH and low oxygen (↓pH x ↓DO, green), and combined elevated temperature, low pH, and low oxygen (↑Temp x ↓pH x ↓DO, brown).

Supplementary Table S2. Summary of the model for the statistical analysis of skeletal growth for *Dendrophyllia cornigera* under the different treatments. SD = standard deviation, SE = standard error, df = degrees of freedom. Significant differences are denoted by *** ($p \leq 0.001$).

Random Effects	Name	Variance	SD
Colony	Intercept	< 0.001	0.010
Aquarium	Intercept	< 0.001	0.007
Residual		< 0.001	0.016

Fixed Effects	Estimate	SE	df	t-value	p	
Intercept	0.032	0.007	16.117	4.261	0.001	***
Treatment ↑Temp	0.001	0.010	13.441	0.054	0.958	
Treatment ↓pH	-0.002	0.010	14.217	-0.220	0.829	
Treatment ↓DO	-0.006	0.010	14.316	-0.573	0.575	
Treatment ↑Temp x ↓pH	0.004	0.010	15.304	0.419	0.681	
Treatment ↑Temp x ↓DO	-0.009	0.010	13.555	-0.957	0.355	
Treatment ↓pH x ↓DO	-0.010	0.010	15.776	-0.946	0.358	
Treatment ↑Temp x ↓pH x ↓DO	0.001	0.010	16.003	0.143	0.888	

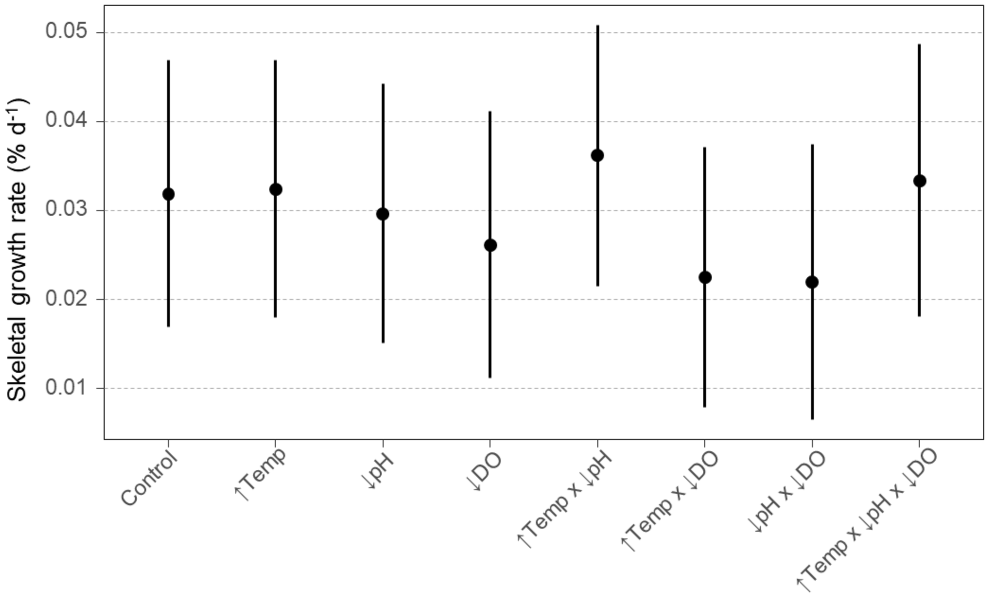

Supplementary Fig. S3. Predicted skeletal growth of *Dendrophyllia cornigera* under the different treatments over nine months, based on the applied linear mixed-effects model. Points indicate the means, and lines indicate the 95% CI for each treatment.

Supplementary Table S3. Summary of the model for the statistical analysis of tissue cover for *Dendrophyllia cornigera* under the different treatments. SD = standard deviation, SE = standard error, df = degrees of freedom.

Random Effects	Name	Variance	SD
Colony	Intercept	< 0.001	0.004
Aquarium	Intercept	< 0.001	< 0.001
Residual		0.001	0.036

Fixed Effects	Estimate	SE	df	t-value	p
Intercept	-0.011	0.013	53.279	-0.832	0.409
Treatment ↑Temp	0.016	0.018	56.030	0.907	0.368
Treatment ↓pH	-0.004	0.018	56.997	-0.206	0.837
Treatment ↓DO	-0.003	0.019	56.561	-0.187	0.853
Treatment ↑Temp x ↓pH	0.005	0.018	54.964	0.303	0.763
Treatment ↑Temp x ↓DO	-0.006	0.018	56.775	-0.321	0.749
Treatment ↓pH x ↓DO	-0.002	0.018	56.713	-0.105	0.916
Treatment ↑Temp x ↓pH x ↓DO	0.026	0.018	56.098	1.466	0.148

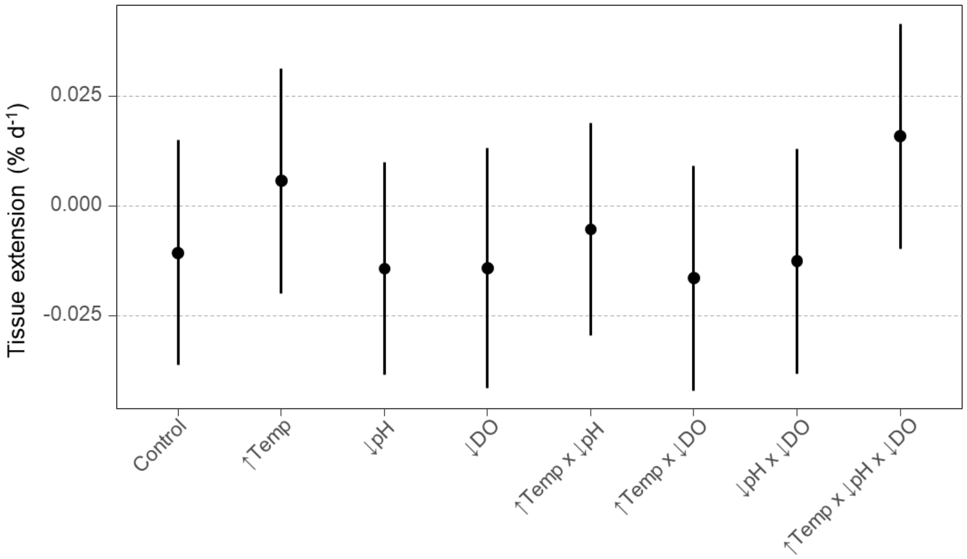

Supplementary Fig. S4. Predicted tissue cover of *Dendrophyllia cornigera* under the different treatments over nine months, based on the applied linear mixed-effects model. Points indicate the means, and lines indicate the 95% CI for each treatment.

Supplementary Table S4. Summary of the model for the statistical analysis of respiration for *Dendrophyllia cornigera* under the different treatments after 6 and 9 months. SD = standard deviation, SE = standard error, df = degrees of freedom. Significant differences are denoted by *** ($p \leq 0.001$).

Random Effects	Name	Variance	SD
Coral ID:Colony	Intercept	0.007	0.085
Coral ID:Aquarium	Intercept	0.001	0.031
Colony	Intercept	0.034	0.186
Aquarium	Intercept	< 0.001	< 0.001
Residual		0.054	0.232

Fixed Effects	Estimate	SE	df	t-value	p	
Intercept	1.462	0.286	69.699	5.110	< 0.001	***
Treatment ↑Temp	-0.406	0.399	66.491	-1.016	0.313	
Treatment ↓pH	0.404	0.405	68.057	0.997	0.322	
Treatment ↓DO	-0.006	0.412	66.398	-0.014	0.989	
Treatment ↑Temp x ↓pH	0.417	0.413	69.863	1.010	0.316	
Treatment ↑Temp x ↓DO	0.383	0.410	67.953	0.934	0.354	
Treatment ↓pH x ↓DO	-0.769	0.414	67.920	-1.857	0.068	
Treatment ↑Temp x ↓pH x ↓DO	-0.143	0.414	67.739	-0.346	0.730	
Time 9 m	-0.070	0.036	58.777	-1.931	0.058	
Time 9 m : Treatment ↑Temp	0.064	0.052	58.777	1.246	0.218	
Time 9 m : Treatment ↓pH	-0.073	0.053	60.438	-1.382	0.172	
Time 9 m : Treatment ↓DO	-0.023	0.053	58.777	-0.440	0.662	
Time 9 m : Treatment ↑Temp x ↓pH	-0.054	0.053	60.371	-1.021	0.311	
Time 9 m : Treatment ↑Temp x ↓DO	-0.056	0.053	60.218	-1.054	0.296	
Time 9 m : Treatment ↓pH x ↓DO	0.090	0.053	58.777	1.686	0.097	
Time 9 m : Treatment ↑Temp x ↓pH x ↓DO	0.025	0.053	58.777	0.465	0.643	

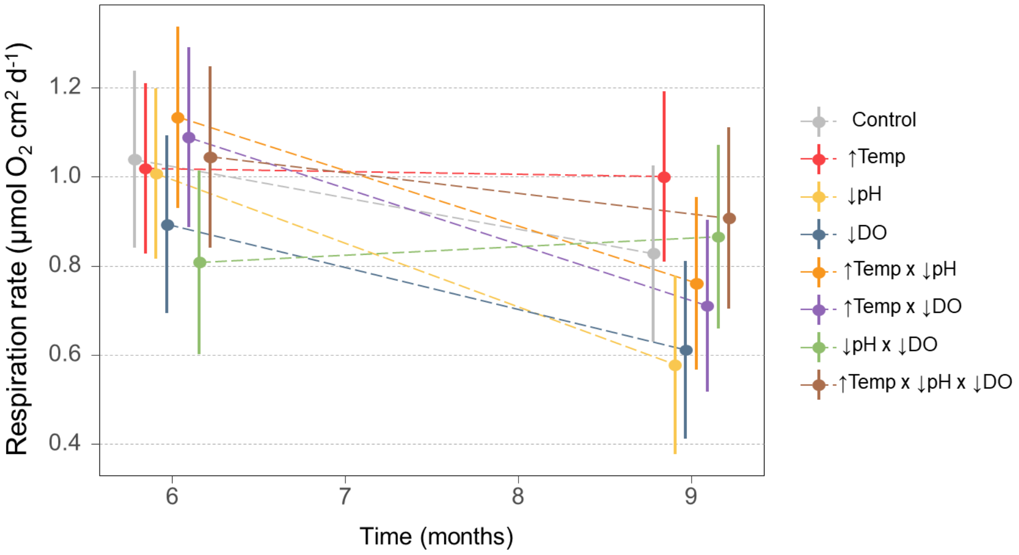

Supplementary Fig. S5. Predicted respiration of *Dendrophyllia cornigera* under the different treatments from six to nine months, based on the applied linear mixed-effects model. Points indicate the means and vertical lines indicate their 95% CI for each treatment. Treatment conditions are: control (grey), elevated temperature (↑Temp, red), low pH (↓pH, yellow), low oxygen (↓DO, blue), combined elevated temperature and low pH (↑Temp x ↓pH, orange), combined elevated temperature and low oxygen (↑Temp x ↓DO, purple), combined low pH and low oxygen (↓pH x ↓DO, green), and combined elevated temperature, low pH, and low oxygen (↑Temp x ↓pH x ↓DO, brown).

Supplementary Table S5. Results of the *post hoc* pair-wise comparisons on the estimated marginal means for respiration of *Dendrophyllia cornigera* under the different treatments and between 6 and 9 months of experimental time. SE = standard error, df = degrees of freedom. Significant differences are denoted by * (p≤ 0.05), ** (p≤ 0.01), *** (p≤ 0.001).

**Contrast**	**Estimate**	**SE**	**df**	**t. ratio**	**p**	
Time: 6 – 9 months						
Control	0.211	0.109	58.315	1.931	0.058	
↑Temp	0.018	0.109	58.315	0.169	0.866	
↓pH	0.429	0.114	61.542	3.764	< 0.001	***
↓DO	0.281	0.116	58.315	2.425	0.018	*
↑Temp x ↓pH	0.373	0.114	61.415	3.262	0.002	**
↑Temp x ↓DO	0.378	0.114	61.110	3.303	0.002	**
↓pH x ↓DO	-0.058	0.116	58.315	-0.496	0.622	
↑Temp x ↓pH x ↓DO	1.137	0.116	58.315	1.181	0.242	
Treatment after 6 months						
Control – ↑Temp	0.020	0.128	26.615	0.160	1.000	
Control – ↓pH	0.032	0.131	26.693	0.242	1.000	
Control – ↓DO	0.146	0.131	27.547	1.112	0.949	
Control – ↑Temp x ↓pH	-0.094	0.141	31.106	-0.670	0.997	
Control – ↑Temp x ↓DO	-0.049	0.130	29.258	-0.379	1.000	
Control – ↓pH x ↓DO	0.231	0.141	31.498	1.643	0.721	
Control – ↑Temp x ↓pH x ↓DO	-0.005	0.139	31.133	-0.038	1.000	
↑Temp – ↓pH	0.011	0.129	26.578	0.089	1.000	
↑Temp – ↓DO	0.126	0.131	29.146	0.957	0.977	
↑Temp – ↑Temp x ↓pH	-0.115	0.136	29.245	-0.840	0.989	
↑Temp – ↑Temp x ↓DO	-0.070	0.132	28.957	-0.529	0.999	
↑Temp – ↓pH x ↓DO	0.211	0.139	32.486	1.516	0.793	
↑Temp – ↑Temp x ↓pH x ↓DO	-0.026	0.137	31.164	-0.187	1.000	
↓pH – ↓DO	0.114	0.126	26.771	0.904	0.983	
↓pH – ↑Temp x ↓pH	-0.126	0.128	26.288	-0.984	0.972	
↓pH – ↑Temp x ↓DO	-0.081	0.132	28.027	-0.615	0.998	
↓pH – ↓pH x ↓DO	0.199	0.133	27.147	1.497	0.802	
↓pH – ↑Temp x ↓pH x ↓DO	-0.037	0.133	28.733	-0.279	1.000	
↓DO – ↑Temp x ↓pH	-0.240	0.136	31.472	-1.773	0.641	
↓DO – ↑Temp x ↓DO	-0.195	0.136	31.837	-1.439	0.832	
↓DO – ↓pH x ↓DO	0.085	0.139	32.245	0.613	0.998	

↓DO – ↑Temp x ↓pH x ↓DO	-0.151	0.135	31.628	-1.123	0.947
↑Temp x ↓pH – ↑Temp x ↓DO	0.045	0.137	29.042	0.328	1.000
↑Temp x ↓pH – ↓pH x ↓DO	0.325	0.141	33.145	2.302	0.322
↑Temp x ↓pH – ↑Temp x ↓pH x ↓DO	0.089	0.140	31.908	0.634	0.998
↑Temp x ↓DO – ↓pH x ↓DO	0.281	0.142	32.852	1.969	0.516
↑Temp x ↓DO – ↑Temp x ↓pH x ↓DO	0.044	0.137	30.276	0.322	1.000
↓pH x ↓DO – ↑Temp x ↓pH x ↓DO	-0.236	0.139	31.708	-1.707	0.683
Treatment after 9 months					
Control – ↑Temp	-0.172	0.128	26.615	-1.351	0.870
Control – ↓pH	0.250	0.135	28.055	1.853	0.592
Control – ↓DO	0.216	0.131	27.547	1.646	0.720
Control – ↑Temp x ↓pH	0.067	0.137	29.632	0.489	1.000
Control – ↑Temp x ↓DO	0.117	0.128	27.112	0.919	0.981
Control – ↓pH x ↓DO	-0.038	0.141	31.498	-0.269	1.000
Control – ↑Temp x ↓pH x ↓DO	-0.080	0.139	31.133	-0.571	0.999
↑Temp – ↓pH	0.422	0.132	27.959	3.187	0.060
↑Temp – ↓DO	0.389	0.131	29.146	2.961	0.096
↑Temp – ↑Temp x ↓pH	0.239	0.133	27.801	1.804	0.623
↑Temp – ↑Temp x ↓DO	0.290	0.129	26.914	2.245	0.358
↑Temp – ↓pH x ↓DO	0.135	0.139	32.486	0.968	0.976
↑Temp – ↑Temp x ↓pH x ↓DO	0.093	0.137	31.164	0.676	0.997
↓pH – ↓DO	-0.034	0.130	28.752	-0.260	1.000
↓pH – ↑Temp x ↓pH	-0.183	0.129	27.873	-1.416	0.842
↓pH – ↑Temp x ↓DO	-0.133	0.133	28.306	-1.000	0.971
↓pH – ↓pH x ↓DO	-0.288	0.138	29.571	-2.083	0.448
↓pH – ↑Temp x ↓pH x ↓DO	-0.329	0.137	31.117	-2.412	0.271
↓DO – ↑Temp x ↓pH	-0.149	0.132	30.142	-1.132	0.944
↓DO – ↑Temp x ↓DO	-0.099	0.133	30.442	-0.744	0.995
↓DO – ↓pH x ↓DO	-0.254	0.139	32.245	-1.829	0.606
↓DO – ↑Temp x ↓pH x ↓DO	-0.296	0.135	31.628	-2.195	0.381
↑Temp x ↓pH – ↑Temp x ↓DO	0.050	0.131	26.536	0.386	1.000
↑Temp x ↓pH – ↓pH x ↓DO	-0.105	0.136	28.967	-0.771	0.993
↑Temp x ↓pH – ↑Temp x ↓pH x ↓DO	-0.147	0.137	30.065	-1.072	0.958
↑Temp x ↓DO – ↓pH x ↓DO	-0.155	0.139	31.330	-1.113	0.949
↑Temp x ↓DO – ↑Temp x ↓pH x ↓DO	-0.197	0.134	29.168	-1.469	0.817
↓pH x ↓DO – ↑Temp x ↓pH x ↓DO	-0.042	0.139	31.708	-0.301	1.000

Supplementary Table S6. Summary of aquaria experiments assessing the single and combined effects of elevated temperature, low pH, and low oxygen in cold-water corals. The study species are *D. cornigera* = *Dendrophyllia cornigera, D. ramea* = *Dendrophyllia ramea, C. huinayensis* = *Caryophyllia huinayensis, C. smithii* = *Caryophyllia smithii, D. dianthus* = *Desmophyllum dianthus, D. pertusum* = *Desmophyllum pertusum, M. oculata* = *Madrepora oculata,* and *S. variabilis* = *Solenosmilia variabilis*. The study areas are NE-A = North-East Atlantic, Med = Mediterranean, SE-P = South-East Pacific, GoM = Gulf of Mexico, NE-P = North-East Pacific, NW-A = North-West Atlantic, SE-A = South-East Atlantic, and SW-P = South-West Pacific. Depth indicates the depth of the collection site in m. Treatments are ↑Temp = elevated temperature, ↓pH = low pH, ↓DO = low oxygen, ↑Temp x ↓pH = combined elevated temperature and low pH, ↑Temp x ↓DO = combined elevated temperature and low oxygen, ↓pH x ↓DO = combined low pH and low oxygen, and ↑Temp x ↓pH x ↓DO = combined elevated temperature, low pH, and low oxygen. The studied response variables are S = survival, G = skeletal growth/calcification, T = tissue cover, and R = Respiration. Significant differences for each treatment and response variable in comparison to the control are expressed as ↑ = Increase, ↓ = Decrease, = = None. Temperature is expressed in ºC. pH units are expressed in total scale at *in situ* temperature (* = free scale). Partial pressure of CO_2 (pCO_2) is expressed in µatm (** = ppm). Ω_{Ar} = saturation state of seawater with respect to aragonite. DO = dissolved oxygen concentration, expressed as mL L^{-1}.

Species	Area	Depth	Duration	Treatment	S	G	T	R	Temperature	pH	pCO_2	Ω_{Ar}	DO	Reference
D. cornigera	NE-A	100–150	9 months	Control					12.4 ± 0.4	7.98 ± 0.04	483 ± 51	1.97 ± 0.15	6.42 ± 0.13	This study
D. cornigera	NE-A	100–150	9 months	↑Temp	=	=	=	=	15.1 ± 0.2	7.94 ± 0.04	546 ± 54	1.99 ± 0.15	6.29 ± 0.13	This study
D. cornigera	NE-A	100–150	9 months	↓pH	=	=	=	=	12.4 ± 0.4	7.69 ± 0.08	1,013 ± 171	1.10 ± 0.19	6.40 ± 0.14	This study
D. cornigera	NE-A	100–150	9 months	↓DO	=	=	=	=	12.3 ± 0.3	8.00 ± 0.04	462 ± 54	2.04 ± 0.15	4.75 ± 0.23	This study
D. cornigera	NE-A	100–150	9 months	↑Temp x ↓pH	=	=	=	=	15.0 ± 0.2	7.68 ± 0.04	1,061 ± 100	1.16 ± 0.09	6.24 ± 0.11	This study
D. cornigera	NE-A	100–150	9 months	↑Temp x ↓DO	=	=	=	=	15.0 ± 0.3	7.95 ± 0.04	527 ± 60	2.04 ± 0.16	4.77 ± 0.19	This study
D. cornigera	NE-A	100–150	9 months	↓pH x ↓DO	=	=	=	=	12.2 ± 0.3	7.67 ± 0.05	1,072 ± 133	1.03 ± 0.11	4.76 ± 0.25	This study

Species	Area	Depth	Duration	Treatment	S	G	T	R	Temperature	pH	pCO_2	Ω_{Ar}	DO	Reference
D. cornigera	NE-A	100–150	9 months	↑Temp x ↓pH x ↓DO	=	=	=	=	15.0 ± 0.3	7.64 ± 0.03	1,159 ± 89	1.07 ± 0.05	4.81 ± 0.30	This study
D. cornigera	Med	300	3 months	Control					12.5					[2]
D. cornigera	Med	300	3 months	↑Temp	=	↑			17.5					[2]
D. cornigera	Med	250	314 d	Control					12	8.10 ± 0.02	379 ± 23**	2.8 ± 0.1		[3]
D. cornigera	Med	250	314 d	↓pH		=			12	7.81 ± 0.03	810 ± 53**	1.6 ± 0.1		[3]
D. cornigera	Med	126	3 months	Control					13.4 ± 0.2	8.07 ± 0.04	399 ± 38	2.72 ± 0.19		[4]
D. cornigera	Med	126	3 months	↓pH	=	=		=	13.4 ± 0.3	7.70 ± 0.06	1,058 ± 168	1.29 ± 0.17		[4]
D. cornigera	Med	180–330	5 months	Control					12					[5]
D. cornigera	Med	180–330	5 months	↑Temp	=	↑	↑	=	16					[5]
D. cornigera	Med	180–330	20 months	Control					12					[6]
D. cornigera	Med	180–330	20 months	↑Temp	=	↓		↑	16					[6]
D. cornigera	Med	180–330	20 months	↑Temp	↓		↓		20					[6]
D. ramea	Med	150	20 months	Control					16					[6]
D. ramea	Med	150	20 months	↑Temp	=	↓		=	20					[6]
D. ramea	Med	150	20 months	↑Temp	=	↓		=	24					[6]
C. huinayensis	SE-P	20–45	6 months	Control					11.2 ± 0.1	8.06 ± 0.03	476 ± 36	2.44 ± 0.16		[7]
C. huinayensis	SE-P	20–45	6 months	↑Temp	↓	↓	↓	↑	15.0 ± 0.1	8.05 ± 0.02	473 ± 23	2.67 ± 0.12		[7]
C. huinayensis	SE-P	20–45	6 months	↓pH	=	=	=	=	11.2 ± 0.1	7.54 ± 0.04	1,725 ± 160	0.82 ± 0.08		[7]

Species	Area	Depth	Duration	Treatment	S	G	T	R	Temperature	pH	pCO_2	Ω_{Ar}	DO	Reference
C. huinayensis	SE-P	20–45	6 months	↑Temp x ↓pH	↓	↓	↓	=	15.1 ± 0.1	7.50 ± 0.04	1,902 ± 186	0.86 ± 0.08		[7]
C. smithii	Med	600	3 months	Control					13.4 ± 0.2	8.07 ± 0.04	399 ± 38	2.72 ± 0.19		[4]
C. smithii	Med	600	3 months	↓pH	=	=		=	13.4 ± 0.3	7.70 ± 0.06	1,058 ± 168	1.29 ± 0.17		[4]
D. dianthus	Med	430	8 months	Control					12	7.96 ± 0.06	380**			[8]
D. dianthus	Med	430	8 months	↑Temp		↓		=	15	7.97 ± 0.04	380**			[8]
D. dianthus	Med	430	8 months	↓pH		=		=	12	7.92 ± 0.06	750**			[8]
D. dianthus	Med	430	8 months	↑Temp x ↓pH		=		↓	15	7.90 ± 0.06	750**			[8]
D. dianthus	Med	250–300	314 d	Control					12	8.10 ± 0.02	379 ± 23**	2.8 ± 0.1		[3]
D. dianthus	Med	250–300	314 d	↓pH		↓			12	7.81 ± 0.03	810 ± 53**	1.6 ± 0.1		[3]
D. dianthus	Med	467–632	3 months	Control					12.5					[2]
D. dianthus	Med	467–632	3 months	↑Temp	=	=			17.5					[2]
D. dianthus	Med	830	3 months	Control					13.4 ± 0.2	8.07 ± 0.04	399 ± 38	2.72 ± 0.19		[4]
D. dianthus	Med	830	3 months	↓pH	=	=		=	13.4 ± 0.3	7.70 ± 0.06	1,058 ± 168	1.29 ± 0.17		[4]
D. dianthus	NE-A	450	8 months	Control					12.5 ± 0.5	8.01 ± 0.06	460.7 ± 22.5	2.19 ± 0.06		[9]
D. dianthus	NE-A	450	8 months	↓pH	=	=		=	12.5 ± 0.5	7.70 ± 0.04	996.9 ± 11.6	1.16 ± 0.01		[9]
D. dianthus	SE-P	145–220	6 months	Control					11.0 ± 0.1	8.12 ± 0.01	360 ± 10	2.79 ± 0.04		[10]
D. dianthus	SE-P	145–220	6 months	↓pH	↓	↓	↓		11.0 ± 0.1	7.25 ± 0.02	3,180 ± 190	0.45 ± 0.02		[10]
D. dianthus	SE-P	145–220	6 months	↓pH	=	↓			11.0 ± 0.1	7.50 ± 0.02	1,730 ± 100	0.79 ± 0.04		[10]

Species	Area	Depth	Duration	Treatment	S	G	T	R	Temperature	pH	pCO_2	Ω_{Ar}	DO	Reference
D. dianthus	SE-P	145–220	6 months	↓pH	=	=			11.1 ± 0.2	7.73 ± 0.05	1,030 ± 140	1.31 ± 0.13		[10]
D. pertusum	GoM	462–465	7 d	Control					8					[11]
D. pertusum	GoM	462–465	7 d	↑Temp	=				15					[11]
D. pertusum	GoM	462–465	7 d	↑Temp	↓				20					[11]
D. pertusum	GoM	462–465	7 d	↑Temp	↓				25					[11]
D. pertusum	GoM	385–550	7 d	Control					8.4 ± 0.2	7.92 ± 0.04		1.53 ± 0.14	6	[12]
D. pertusum	GoM	385–550	7 d	↑Temp	↓				9.9 ± 0.5	7.92 ± 0.04		1.54 ± 0.12	6	[12]
D. pertusum	GoM	385–550	7 d	↑Temp	↓				11.9 ± 0.3	7.89 ± 0.07		1.68 ± 0.22	6	[12]
D. pertusum	GoM	385–550	7 d	↑Temp	↓				14.0 ± 0.3	7.93 ± 0.03		1.92 ± 0.14	6	[12]
D. pertusum	GoM	385–550	7 d	↑Temp	↓				16.0 ± 0.5	7.95 ± 0.04		2.08 ± 0.18	6	[12]
D. pertusum	GoM	385–400	7 d	Control					8.6 ± 0.5	7.90 ± 0.06		1.47 ± 0.17	6	[12]
D. pertusum	GoM	385–400	7 d	↓pH	=	↓			7.8 ± 0.4	7.80 ± 0.07		1.18 ± 0.18	6	[12]
D. pertusum	GoM	385–400	7 d	↓pH	=	↓			8.4 ± 0.5	7.67 ± 0.16		0.97 ± 0.40	6	[12]
D. pertusum	GoM	390–550	7 d	Control					8.8 ± 0.3	8.07 ± 0.06		3.23 ± 0.38	2.92 ± 0.21	[12]
D. pertusum	GoM	390–550	7 d	↓DO	↓				8.5 ± 0.5	8.28 ± 0.12		4.88 ± 1.12	1.57 ± 0.28	[12]
D. pertusum	GoM	450–500	2 weeks	Control					8.1 ± 0.2	7.92 ± 0.03	552 ± 42	1.51 ± 0.10		[13]
D. pertusum	GoM	450–500	2 weeks	↓pH	=	↓	=	↓	8.1 ± 0.2	7.62 ± 0.03	1,165 ± 76	0.80 ± 0.05		[13]
D. pertusum	GoM	450–500	2 weeks	↓pH	=	↓	=	↓	8.1 ± 0.2	7.76 ± 0.03	831 ± 54	1.07 ± 0.07		[13]

Species	Area	Depth	Duration	Treatment	S	G	T	R	Temperature	pH	*p*CO_2	Ω_{Ar}	DO	Reference
D. pertusum	GoM	451–494	2 weeks	Control					9.0 ± 0.1	7.91 ± 0.07	545 ± 14	1.49 ± 0.04		[14]
D. pertusum	GoM	451–494	2 weeks	↓pH		=			8.2 ± 0.1	7.74 ± 0.02	833 ± 31	0.99 ± 0.06		[14]
D. pertusum	GoM	451–494	2 weeks	↓pH		=			8.6 ± 0.1	7.62 ± 0.03	1,154 ± 88	0.81 ± 0.06		[14]
D. pertusum	GoM	451–494	6 months	Control					8.0 ± 0.1	7.91 ± 0.04	560 ± 75	1.43 ± 0.14		[14]
D. pertusum	GoM	451–494	6 months	↓pH		↓			7.8 ± 0.1	7.65 ± 0.07	1,161 ± 216	0.90 ± 0.14		[14]
D. pertusum	Med	250–300	6 months	Control					12	8.10 ± 0.02	384 ± 23**	2.8 ± 0.1		[15]
D. pertusum	Med	250–300	6 months	↓pH		=			12	7.81 ± 0.03	809 ± 61**	1.6 ± 0.1		[15]
D. pertusum	Med	540	6 months	Control					13					[16]
D. pertusum	Med	540	6 months	↑Temp	=	=			15					[16]
D. pertusum	Med	540	6 months	↑Temp	↓	=			17					[16]
D. pertusum	Med	260–500	3 months	Control					13.0 ± 0.1	8.13 ± 0.02	350 ± 20.3	3.05 ± 0.1		[17]
D. pertusum	Med	260–500	3 months	↓pH				=	13.0 ± 0.1	7.69 ± 0.02	1,108 ± 58.3	1.25 ± 0.05		[17]
D. pertusum	Med	260–500	3 months	↓pH				=	13.0 ± 0.1	7.80 ± 0.03	826 ± 68.9	1.61 ± 0.11		[17]
D. pertusum	Med	260–500	3 months	↓pH				=	13.0 ± 0.1	8.00 ± 0.02	497 ± 27.5	2.37 ± 0.10		[17]
D. pertusum	Med	260–500	9 months	Control					11.0 ± 0.1	8.10 ± 0.02	379 ± 10	2.80 ± 0.11		[18]
D. pertusum	Med	260–500	9 months	↓pH		=			11.0 ± 0.1	7.73 ± 0.02	969 ± 25	1.30 ± 0.08		[18]
D. pertusum	Med	260–500	9 months	↓pH		=			11.0 ± 0.1	7.85 ± 0.01	713 ± 19	1.70 ± 0.05		[18]
D. pertusum	Med	260–500	9 months	↓pH		=			11.0 ± 0.1	8.00 ± 0.01	489 ± 13	2.40 ± 0.06		[18]

Species	Area	Depth	Duration	Treatment	S	G	T	R	Temperature	pH	$p\mathrm{CO_2}$	Ω_{Ar}	DO	Reference
D. pertusum	NE-A	130	96 h	Control					9				6.2–8.2	[19]
D. pertusum	NE-A	130	96 h	↑Temp				↑	11				6.0–7.8	[19]
D. pertusum	NE-A	141–167	21 d	Control					9.5	8.06 ± 0.01	380**	2.02 ± 0.01		[20]
D. pertusum	NE-A	141–167	21 d	↓pH		=		↓	9.5	7.77 ± 0.01	750**	1.03 ± 0.01		[20]
D. pertusum	NE-A	141–167	12 months	Control					9	8.01 ± 0.02	489.4 ± 58.4	1.56 ± 0.15		[21]
D. pertusum	NE-A	141–167	12 months	↑Temp	=	=		↓	12	8.00 ± 0.03	562 ± 44.1	1.49 ± 0.20		[21]
D. pertusum	NE-A	141–167	12 months	↓pH	=	=		=	9	7.78 ± 0.05	988 ± 139.6	0.76 ± 0.10		[21]
D. pertusum	NE-A	141–167	12 months	↓pH	=	=		=	9	7.89 ± 0.02	802.5 ± 121	1.09 ± 0.16		[21]
D. pertusum	NE-A	141–167	12 months	↑Temp x ↓pH	=	=		=	12	7.91 ± 0.04	773.4 ± 88.1	1.19 ± 0.13		[21]
D. pertusum	NE-A	165–280	58 h	Control					7					[22]
D. pertusum	NE-A	165–280	58 h	↑Temp	=			↑	9					[22]
D. pertusum	NE-A	165–280	58 h	↑Temp	=			↑	11					[22]
D. pertusum	NE-A	165–280	58 h	↑Temp	=			↑	13					[22]
D. pertusum	NE-A	165–280	58 h	↑Temp	=			↑	15					[22]
D. pertusum	NE-A	800	8 weeks	Control					10					[23]
D. pertusum	NE-A	800	8 weeks	Temp	↓	=			13					[23]
D. pertusum	NE-A	800	8 weeks	Temp	↓	=			15					[23]
D. pertusum	NE-A	145–220	6 months	Control					8		400			[24]

Species	Area	Depth	Duration	Treatment	S	G	T	R	Temperature	pH	*p*CO_2	Ω_{Ar}	DO	Reference
D. pertusum	NE-A	145–220	6 months	↑Temp	=	↑			12		400			[24]
D. pertusum	NE-A	145–220	6 months	↓pH	=	↓			8		800			[24]
D. pertusum	NE-A	145–220	6 months	↑Temp x ↓pH	=	=			12		800			[24]
D. pertusum	NE-A	100–285	6 months	Control					7–7.5 ± 0.5	7.94 ± 0.06*	604 ± 105	1.37 ± 0.16		[25]
D. pertusum	NE-A	100–285	6 months	↓pH		=		=	7–7.5 ± 0.5	± 0.06*	982 ± 146	0.93 ± 0.10		[25]
D. pertusum	NE-A	100–285	6 months	↓pH		=		=	7–7.5 ± 0.5	7.83 ± 0.05*	778 ± 112	1.03 ± 0.09		[25]
D. pertusum	NE-A	150–230	13 months	Control					8.0 ± 0.1	8.01 ± 0.02	467 ± 31	1.94 ± 0.08		[26]
D. pertusum	NE-A	150–230	13 months	↑Temp	=	↑		↑	15.1 ± 0.1	8.01 ± 0.01	467 ± 8	2.41 ± 0.07		[26]
D. pertusum	NE-A	150–230	13 months	↓pH	=	↓		↓	8.2 ± 0.2	7.57 ± 0.06	1,586 ± 252	0.84 ± 0.08		[26]
D. pertusum	NE-A	150–230	13 months	↑Temp x ↓pH	=	=		↑	15 ± 0.2	7.54 ± 0.01	1,601 ± 56	0.97 ± 0.03		[26]
D. pertusum	NE-A	106–112	24 h	Control					7.5	8.10	386**	1.89		[27]
D. pertusum	NE-A	106–112	24 h	↓pH		↓			7.5	7.76	791**	0.97		[27]
D. pertusum	NE-A	106–112	24 h	↓pH		=			7.5	7.91	544**	1.38		[27]
D. pertusum	NE-A	100	2 weeks	Control					7.9 ± 0.1	7.91 ± 0.03	579 ± 41	1.38 ± 0.08		[13]
D. pertusum	NE-A	100	2 weeks	↓pH	=	=	=	↑	7.9 ± 0.1	7.6 ± 0.03	1,208 ± 132	0.74 ± 0.07		[13]
D. pertusum	NE-A	100	2 weeks	↓pH	=	=	=	↑	7.9 ± 0.1	7.76 ± 0.03	845 ± 61	1.00 ± 0.05		[13]
D. pertusum	NE-P	300	21 d	Control					9.5	7.89 ± 0.04	580 ± 8	1.47 ± 0.03		[28]
D. pertusum	NE-P	300	21 d	↓pH	=	↓			9.5	7.62 ± 0.06	1,159 ± 34	0.83 ± 0.03		[28]

Species	Area	Depth	Duration	Treatment	S	G	T	R	Temperature	pH	pCO_2	Ω_{Ar}	DO	Reference
D. pertusum	NW-A	650–850	7 d	Control					8.3 ± 0.1	7.95 ± 0.03				[29]
D. pertusum	NW-A	650–850	7 d	↑Temp				↑	14.2 ± 0.1	7.95 ± 0.03				[29]
D. pertusum	SE-A	480–500	7 d	Control					8.0 ± 0.3				6.1 ± 0.6	[30]
D. pertusum	SE-A	480–500	7 d	↓DO				=	8.0 ± 0.3				1.4 ± 0.5	[30]
M. oculata	Med	434	2 weeks	Control					13.5		438 ± 19	1.92		[31]
M. oculata	Med	434	2 weeks	↓pH		=		=	13.6		805 ± 51	1.00		[31]
M. oculata	Med	434	2 weeks	↓pH		↓		=	13.6		1,638 ± 62	0.63		[31]
M. oculata	Med	434	2 weeks	↓pH		↓		=	13.6		1,725 ± 51	0.71		[31]
M. oculata	Med	250–300	6 months	Control					12	8.10 ± 0.02	379 ± 23**	2.8 ± 0.1		[15]
M. oculata	Med	250–300	6 months	↓pH		=			12	7.81 ± 0.03	810 ± 53**	1.6 ± 0.1		[15]
M. oculata	Med	540	6 months	Control					13					[16]
M. oculata	Med	540	6 months	↑Temp	=	=			15					[16]
M. oculata	Med	540	6 months	↑Temp	↓	=			17					[16]
M. oculata	Med	260–500	3 months	Control					13.0 ± 0.1	8.13 ± 0.02	350 ± 20.3	3.05 ± 0.10		[17]
M. oculata	Med	260–500	3 months	↓pH				=	13.0 ± 0.1	7.69 ± 0.02	1,108 ± 58.3	1.25 ± 0.05		[17]
M. oculata	Med	260–500	3 months	↓pH				=	13.0 ± 0.1	7.80 ± 0.03	826 ± 68.9	1.61 ± 0.11		[17]
M. oculata	Med	260–500	3 months	↓pH				=	13.0 ± 0.1	8.00 ± 0.02	497 ± 27.5	2.37 ± 0.10		[17]
M. oculata	Med	260–500	9 months	Control					11.0 ± 0.1	8.10 ± 0.02	380 ± 12	2.90 ± 0.11		[18]

Species	Area	Depth	Duration	Treatment	S	G	T	R	Temperature	pH	pCO_2	Ω_{Ar}	DO	Reference
M. oculata	Med	260–500	9 months	↓pH		=			11.0 ± 0.1	7.74 ± 0.02	947 ± 20	1.40 ± 0.06		[18]
M. oculata	Med	260–500	9 months	↓pH		=			11.0 ± 0.1	7.86 ± 0.02	707 ± 52	1.80 ± 0.06		[18]
M. oculata	Med	260–500	9 months	↓pH		=			11.0 ± 0.1	8.00 ± 0.04	495 ± 50	2.30 ± 0.21		[18]
M. oculata	Med	500	24 h	Control					12.5 ± 0.5		445	2.6		[32]
M. oculata	Med	500	24 h	↓pH		=			12.5 ± 0.5		867	1.9		[32]
S. variabilis	SW-P	1220–1370	12 months	Control					3.5	7.87 ± 0.00	591.9 ± 7.0	1.11 ± 0.02		[33]
S. variabilis	SW-P	1220–1370	12 months	↓pH	=	=	↓	=	3.5	7.65 ± 0.00	1,017.5 ± 15.7	0.69 ± 0.01		[33]

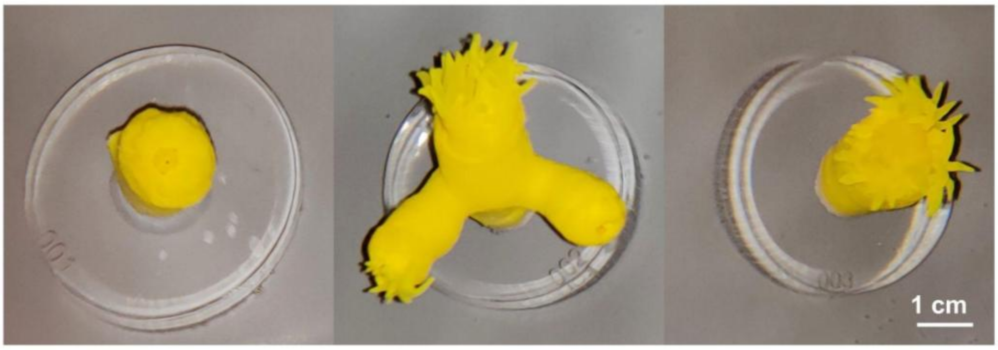

Supplementary Fig. S6. *Dendrophyllia cornigera* nubbins attached to numbered methacrylate bases for the study.

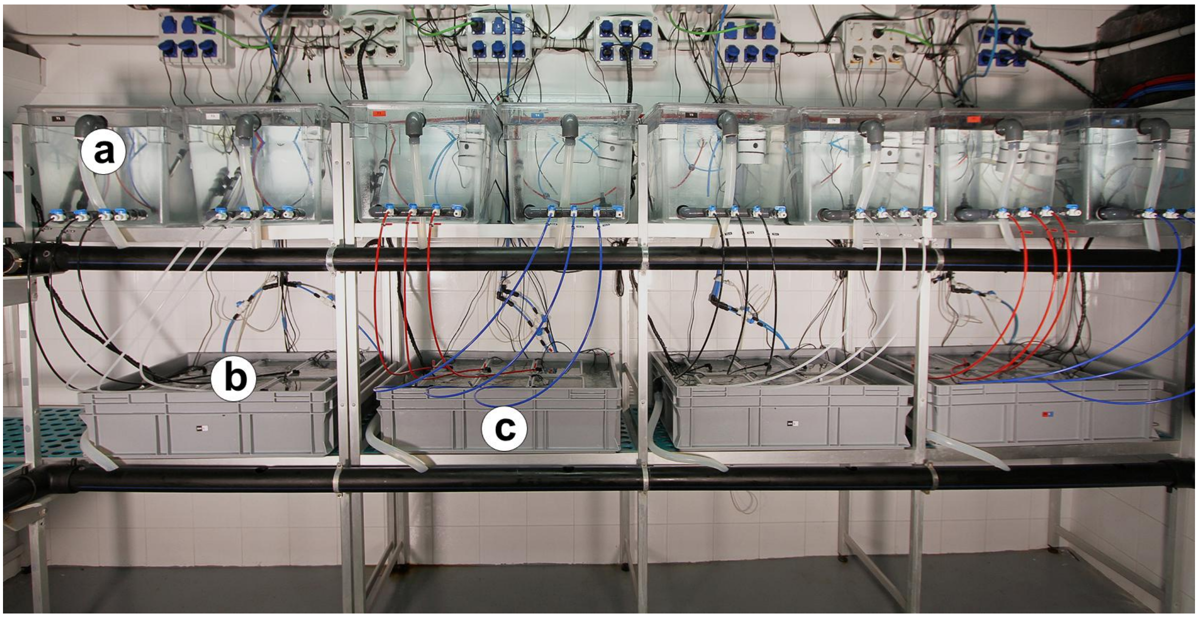

Supplementary Fig. S7. Experimental set-up. Top shelf contains header tanks (**a**) (each assigned to a specific treatment) which supply seawater to three experimental aquaria in the bottom shelf (**b**). Water baths containing several experimental aquaria were used to optimise temperature control (**c**). The experimental set-up is described in detail in Gutiérrez-Zárate et al.[34].

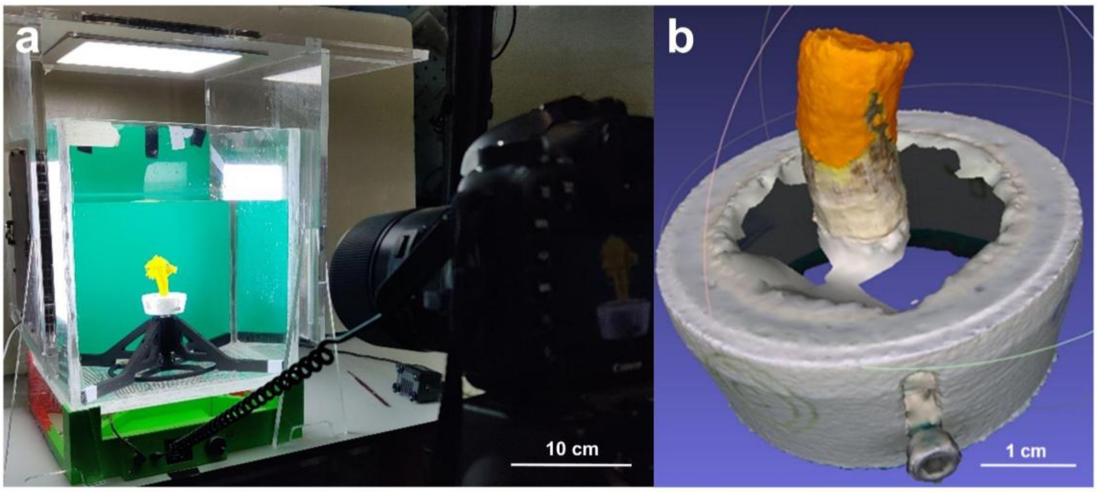

Supplementary Fig. S8. Quantification of tissue surface area of *Dendrophyllia cornigera* nubbins during the experiment: (**a**) Photogrammetry setup including a 18-L aquarium, a rotating system placed above and below the aquarium to rotate the coral nubbin, and a camera to capture photos after every turn; (**b**) 3D model of a nubbin and selection of the tissue surface (highlighted in orange) to quantify its area using MeshLab software [35]. The photogrammetry set-up is described in detail in Romo et al. [36].

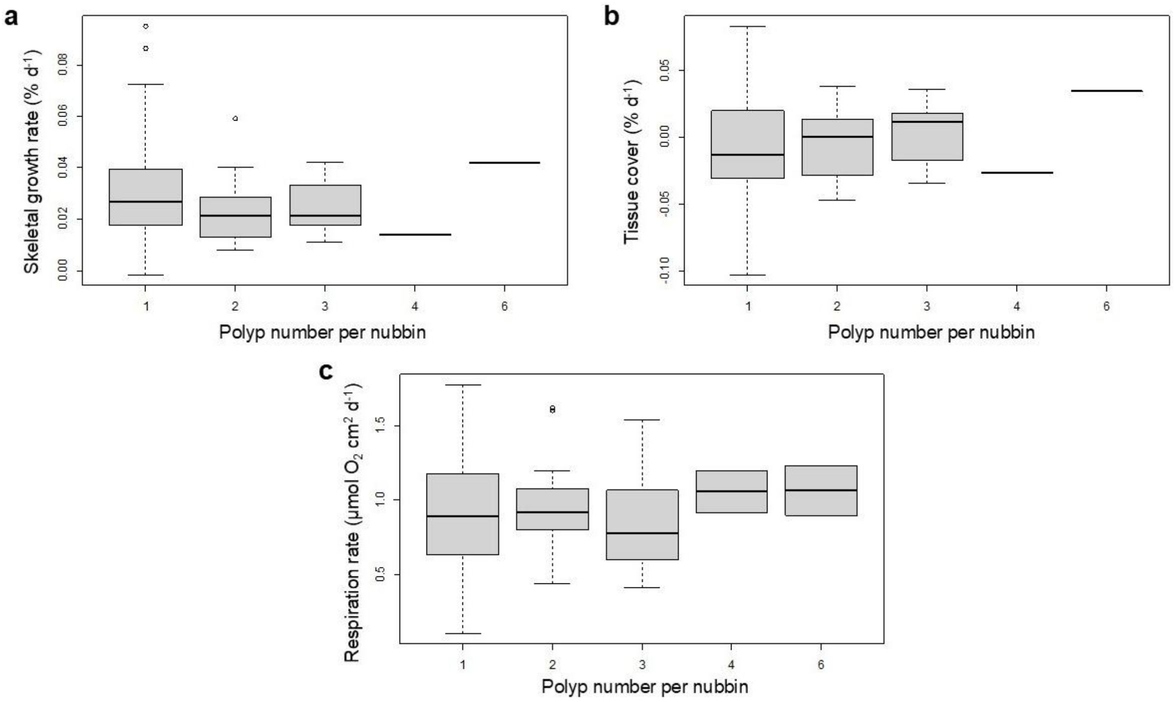

Supplementary Fig. S9. Response variables of *D. cornigera* based on polyp number per nubbin. Response variables are: (**a**) skeletal growth, (**b**) tissue cover and (**c**) respiration rates.

REFERENCES

1. Orejas, C. *et al.* Long-term growth rates of four Mediterranean cold-water coral species maintained in aquaria. *Marine Ecology Progress Series* **429,** 57–65 (2011).
2. Naumann, M. S., Orejas, C. & Ferrier-Pagès, C. High thermal tolerance of two Mediterranean cold-water coral species maintained in aquaria. *Coral Reefs* **32,** 749–754 (2013).
3. Movilla, J. *et al.* Differential response of two Mediterranean cold-water coral species to ocean acidification. *Coral Reefs* **33,** 675–686 (2014).
4. Rodolfo-Metalpa, R. *et al.* Calcification is not the Achilles' heel of cold-water corals in an acidifying ocean. *Global Change Biology* **21,** 2238–2248 (2015).
5. Gori, A., Reynaud, S., Orejas, C., Gili, J.-M. & Ferrier-Pagès, C. Physiological performance of the cold-water coral *Dendrophyllia cornigera* reveals its preference for temperate environments. *Coral Reefs* **33,** 665–674 (2014).
6. Reynaud, S. *et al.* Dendrophylliidae cold-water corals in a warm ocean: The effect of exposure duration on their physiological response. *Deep Sea Research Part II: Topical Studies in Oceanography* **193,** 104962 (2021).
7. Beck, K. K. *et al.* Ontogenetic differences in the response of the cold-water coral *Caryophyllia huinayensis* to ocean acidification, warming and food availability. *Science of The Total Environment* **900,** 165565 (2023).
8. Gori, A. *et al.* Physiological response of the cold-water coral *Desmophyllum dianthus* to thermal stress and ocean acidification. *PeerJ* **4,** e1606 (2016).
9. Carreiro-Silva, M. *et al.* Molecular mechanisms underlying the physiological responses of the cold-water coral *Desmophyllum dianthus* to ocean acidification. *Coral Reefs* **33,** 465–476 (2014).
10. Martínez-Dios, A. *et al.* Effects of low pH and feeding on calcification rates of the cold-water coral *Desmophyllum dianthus*. *PeerJ* **8,** e8236 (2020).

11. Brooke, S., Ross, S. W., Bane, J. M., Seim, H. E. & Young, C. M. Temperature tolerance of the deep-sea coral *Lophelia pertusa* from the southeastern United States. *Deep Sea Research Part II: Topical Studies in Oceanography* **92,** 240–248 (2013).

12. Lunden, J. J., McNicholl, C. G., Sears, C. R., Morrison, C. L. & Cordes, E. E. Acute survivorship of the deep-sea coral *Lophelia pertusa* from the Gulf of Mexico under acidification, warming, and deoxygenation. *Front. Mar. Sci.* **1,** (2014).

13. Georgian, S. E. *et al.* Biogeographic variability in the physiological response of the cold-water coral *Lophelia pertusa* to ocean acidification. *Marine Ecology* **37,** 1345–1359 (2016).

14. Kurman, M. D., Gómez, C. E., Georgian, S. E., Lunden, J. J. & Cordes, E. E. Intra-Specific Variation Reveals Potential for Adaptation to Ocean Acidification in a Cold-Water Coral from the Gulf of Mexico. *Frontiers in Marine Science* **4,** (2017).

15. Movilla, J. *et al.* Resistance of Two Mediterranean Cold-Water Coral Species to Low-pH Conditions. *Water* **6,** 59–67 (2014).

16. Chapron, L. *et al.* Resilience of cold-water coral holobionts to thermal stress. *Proceedings of the Royal Society B: Biological Sciences* **288,** 20212117 (2021).

17. Maier, C. *et al.* Respiration of Mediterranean cold-water corals is not affected by ocean acidification as projected for the end of the century. *Biogeosciences* **10,** 5671–5680 (2013).

18. Maier, C. *et al.* End of the Century pCO_2 Levels Do Not Impact Calcification in Mediterranean Cold-Water Corals. *PLOS ONE* **8,** e62655 (2013).

19. Dodds, L. A., Roberts, J. M., Taylor, A. C. & Marubini, F. Metabolic tolerance of the cold-water coral *Lophelia pertusa* (Scleractinia) to temperature and dissolved oxygen change. *Journal of Experimental Marine Biology and Ecology* **349,** 205–214 (2007).

20. Hennige, S. J. *et al.* Short-term metabolic and growth responses of the cold-water coral *Lophelia pertusa* to ocean acidification. *Deep Sea Research Part II: Topical Studies in Oceanography* **99,** 27–35 (2014).

21. Hennige, S. J. *et al.* Hidden impacts of ocean acidification to live and dead coral framework. *Proceedings of the Royal Society B: Biological Sciences* **282,** 20150990 (2015).

22. Dorey, N., Gjelsvik, Ø., Kutti, T. & Büscher, J. V. Broad Thermal Tolerance in the Cold-Water Coral *Lophelia pertusa* From Arctic and Boreal Reefs. *Frontiers in Physiology* **10,** (2020).

23. Chemel, M. *et al.* Cold-water coral mortality under ocean warming is associated with pathogenic bacteria. *Environmental Microbiome* **19,** 76 (2024).

24. Büscher, J. V., Form, A. U. & Riebesell, U. Interactive Effects of Ocean Acidification and Warming on Growth, Fitness and Survival of the Cold-Water Coral *Lophelia pertusa* under Different Food Availabilities. *Front. Mar. Sci.* **4,** (2017).

25. Form, A. U. & Riebesell, U. Acclimation to ocean acidification during long-term CO_2 exposure in the cold-water coral *Lophelia pertusa*. *Global Change Biology* **18,** 843–853 (2012).

26. Büscher, J. V., Form, A. U., Wisshak, M., Kiko, R. & Riebesell, U. Cold-water coral ecosystems under future ocean change: Live coral performance vs. framework dissolution and bioerosion. *Limnology and Oceanography* **67,** 2497–2515 (2022).

27. Maier, C., Hegeman, J., Weinbauer, M. G. & Gattuso, J.-P. Calcification of the cold-water coral *Lophelia pertusa,* under ambient and reduced pH. *Biogeosciences* **6,** 1671–1680 (2009).

28. Gómez, C. E., Wickes, L., Deegan, D., Etnoyer, P. J. & Cordes, E. E. Growth and feeding of deep-sea coral *Lophelia pertusa* from the California margin under simulated ocean acidification conditions. *PeerJ* **6,** e5671 (2018).

29. Gómez, C. E. *et al.* Natural variability in seawater temperature compromises the metabolic performance of a reef-forming cold-water coral with implications for vulnerability to ongoing global change. *Coral Reefs* (2022). doi:10.1007/s00338-022-02267-2

30. Gori, A. *et al.* Natural hypoxic conditions do not affect the respiration rates of the cold-water coral *Desmophyllum pertusum* (*Lophelia pertusa*) living in the Angola margin (Southeastern Atlantic Ocean). *Deep Sea Research Part I: Oceanographic Research Papers* **197,** 104052 (2023).

31. Maier, C. *et al.* Effects of elevated pCO_2 and feeding on net calcification and energy budget of the Mediterranean cold-water coral *Madrepora oculata*. *Journal of Experimental Biology* **219,** 3208–3217 (2016).

32. Maier, C., Watremez, P., Taviani, M., Weinbauer, M. G. & Gattuso, J. P. Calcification rates and the effect of ocean acidification on Mediterranean cold-water corals. *Proceedings of the Royal Society B: Biological Sciences* **279,** 1716–1723 (2012).

33. Gammon, M. J., Tracey, D. M., Marriott, P. M., Cummings, V. J. & Davy, S. K. The physiological response of the deep-sea coral *Solenosmilia variabilis* to ocean acidification. *PeerJ* **6,** e5236 (2018).

34. Gutiérrez-Zárate, C. *et al.* An aquaria set-up for long-term, multiple-stressor research in marine organisms. *Methods in Ecology and Evolution* 1–13 (2025). doi:10.1111/2041-210X.14488

35. Cignoni, P. *et al.* Meshlab: an open-source mesh processing tool. in *Eurographics Italian Chapter Conference* **2008,** 129–136 (2008).

36. Romo, A. *et al.* A cost-effective, open-source laboratory system for 3D photogrammetric analysis of corals. *Deep Sea Research Part II: Topical Studies in Oceanography* **223,** 105525 (2025).
